# Medical Treatment of Cushing's Disease: An Overview of the Current and Recent Clinical Trials

**DOI:** 10.3389/fendo.2020.00648

**Published:** 2020-12-08

**Authors:** Rosario Pivonello, Rosario Ferrigno, Maria Cristina De Martino, Chiara Simeoli, Nicola Di Paola, Claudia Pivonello, Livia Barba, Mariarosaria Negri, Cristina De Angelis, Annamaria Colao

**Affiliations:** ^1^Dipartimento di Medicina Clinica e Chirurgia, Sezione di Endocrinologia, Università Federico II di Napoli, Naples, Italy; ^2^UNESCO Chair for Health Education and Sustainable Development, Federico II University, Naples, Italy

**Keywords:** clinical trials, Cushing's disease, hypercortisolism, experimental therapy, pituitary tumor, ACTH, cortisol

## Abstract

Cushing's disease (CD) is a serious endocrine disorder characterized by chronic hypercortisolism, or Cushing's syndrome (CS), caused by a corticotroph pituitary tumor, which induces an excessive adrenocorticotropic hormone (ACTH) and consequently cortisol secretion. CD presents a severe clinical burden, with impairment of the quality of life and increase in mortality. Pituitary surgery represents the first-line therapy, but it is non-curative in one third of patients, requiring additional treatments. Among second-line treatments, medical therapy is gradually gaining importance, although the current medical treatments are unable to reach optimal efficacy and safety profile. Therefore, new drugs and new formulations of presently available drugs are currently under clinical investigation in international clinical trials, in order to assess their efficacy and safety in CD, or in the general population of CS. Among pituitary-directed agents, pasireotide, in the twice-daily subcutaneous formulation, has been demonstrated to be an effective treatment both in clinical trials and in real-world studies, and extension studies of the phase II and III clinical trials reported evidence of long-term efficacy with general good safety profile, although associated with frequent hyperglycemia, which requires monitoring of glucose metabolism. Moreover, the most recent once-monthly intramuscular formulation, pasireotide long-acting release (LAR), showed similar efficacy and safety, but associated with potential better compliance profile in CD. Roscovitine is an experimental drug currently under investigation. Among adrenal-directed agents, metyrapone is the only historical agent currently under investigation in a prospective, multicenter, international clinical trial, that would likely clarify its efficacy and safety in a large population of patients with CS. Osilodrostat, a novel agent with a mechanism of action similar to metyrapone, seems to offer a rapid, sustained, and effective disease control of CD, according to recently completed clinical trials, whereas levoketoconazole, a different chemical formulation of the historical agent ketoconazole, is still under investigation in clinical trials, with preliminary evidences showing an effective and safe control of CS. ATR-101 is an experimental drug currently under investigation. Among glucocorticoid receptor-directed drugs, mifepristone has been demonstrated to improve clinical syndrome and comorbidities, especially hypertension and impairment of glucose metabolism, but the occurrence of hypokalemia and in women uterine disorders, due to the concomitant action on progestin receptor, requires caution, whereas the preliminary evidence on relacorilant, characterized by high selectivity for glucocorticoid receptor, suggested good efficacy in the control of hypertension and impairment of glucose metabolism, as well as a good safety profile, in CS. Finally, a limited experience has demonstrated that combination therapy might be an interesting approach in the management of CD. The current review provides a summary of the available evidences from current and recent clinical trials on CD, with a specific focus on preliminary data.

## Introduction

Cushing's disease (CD) is a rare and serious endocrine disorder characterized by excessive adrenocorticotropic hormone (ACTH) secretion by a corticotroph pituitary tumor, consequently driving an excessive cortisol secretion by the adrenal glands. CD represents the most frequent cause of the endogenous type of Cushing's syndrome (CS), accounting for about 70% of cases of CS (1, 2). Although characterized by specific clinical features, including moon face, facial plethora, striae rubrae, and supraclavicular and dorsal fat pads, CD is generally associated with a clinical syndrome which resembles common clinical conditions, and is associated with comorbidities, including visceral obesity, hypertension, diabetes, and dyslipidemia, largely overlapping with features of the most common metabolic syndrome, although it is often complicated with reproductive and sexual disturbances, as well as neuropsychiatric and skeletal disorders (2). Therefore, CD is usually characterized by a relevant diagnostic delay, which contributes to development of multiple and severe comorbidities, impairment of quality of life, and increased mortality, compared with healthy population (2). The serious clinical burden of CD suggests, after the diagnosis, a prompt and efficacious treatment (3, 4). The treatment of CD has the main goals to remove or control the pituitary tumor, but mainly to restore normal cortisol secretion, revert clinical syndrome and comorbidities, and normalize mortality (3, 4). Surgical removal of the pituitary tumor is the current first-line therapeutic option, but around 20% of patients do not experience remission after pituitary surgery, and around 15% of patients with apparent remission present recurrences after an initially successful pituitary surgery, even many years after the remission, with an increasing rate over time; this evidence implies that at least one third of patients with CD is not cured by pituitary surgery and requires additional treatments (4). Moreover, a subgroup of patients is not eligible for surgery because of clinical features, mainly severe comorbidities, or tumor features, mainly large size or difficult location, or alternatively refuses or prefers to differ surgery for personal reasons. In these situations of failure or refusal of pituitary surgery, or non-eligibility for pituitary surgery, different treatments, including repeat pituitary surgery, pituitary radiotherapy, bilateral adrenalectomy, and medical therapy, are considered as additional second-line, or alternative first-line treatments (3, 4). Medical therapy has been gradually gaining importance in the landscape of CD treatment, mainly because of the growing evidences about the efficacy and safety of either old or new agents, allowing a patient-tailored approach (4). Three main drug categories may be actually identified: pituitary-directed drugs, including pasireotide and cabergoline; adrenal-directed drugs, or steroidogenesis inhibitors, including ketoconazole, metyrapone, and mitotane; and glucocorticoid receptor (GR)-directed drugs, or GR antagonists, mainly represented by mifepristone (3, 4). However, none of these compounds is currently able to completely reach the therapeutic goals of treatment for CD (4). Therefore, scientific research is focusing on the identification of new drugs or new formulations of currently used drugs, potentially useful in the treatment of CD, in order to increase the efficacy of medical treatment on clinical, hormonal, and tumor control, as well as to improve the pharmacological safety profile in CD (5). The current review aims to summarize the presently available evidences from currently on-going and recently completed clinical trials on CD, offering a summary of either definitive reports published in scientific literature or preliminary data presented at scientific meetings. In particular, a summary of preliminary data may be useful, especially for clinicians not involved in clinical trials or with difficult access to unpublished data. Indeed, the publication of definitive results of clinical trials, which are generally large multicenter studies, usually occurs months, or even years, after study completion, mainly due to the large amount of data to be properly collected, verified, corrected, and analyzed, before the production of the final report, generally subjected to strict and complex administrative procedures. Therefore, the summary of preliminary data reported in the current review could help clinicians in professional update, while waiting for publication of definitive results of the clinical trials.

**Figure 1:**
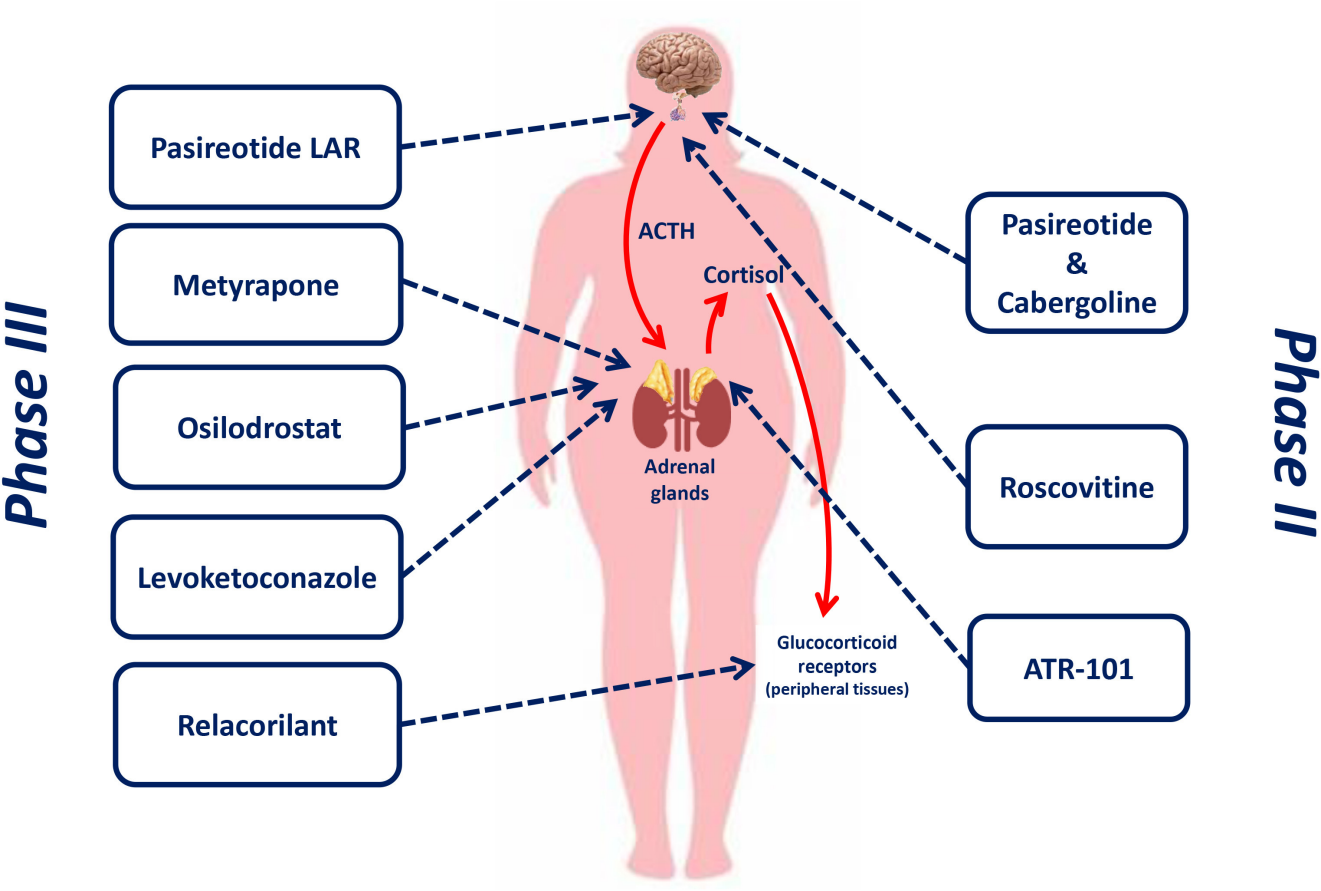
Therapeutic targets of drugs currently or recently investigated in phase II and phase III clinical trials for the treatment of Cushing's disease.

Figure 1 shows the therapeutic targets of drugs currently or recently investigated in phase II and phase III clinical trials for the treatment of CD.

Table 1 provides a summary of the available data regarding drugs evaluated in recent clinical trials or under investigation, registered in clinicaltrials.gov, in CD, and CS, in terms of administration route and timing, available dosages, remission rate, and safety profile, including adverse events (AEs).

**Table 1:** Summary of the available data regarding drugs evaluated in recent clinical trials or under investigation, registered in clinicaltrials.gov, in Cushing's disease and Cushing's syndrome in terms of administration route and timing, available dosages, remission rate, and safety profile.

**Investigation drug**	**Route of administration**	**Timing of administration**	**Available dosages**	Remission rate*	**Principal adverse events**
**PITUITARY-DIRECTED AGENTS**					
Pasireotide	Subcutaneous	Twice daily	300 μg; 600 μg; 900 μg	17.2–81.8%	Hyperglycemia-related AEs (68.4–93.8%); diarrhea (43.6–68.4%); nausea (23.1–68.8%)
Pasireotide LAR	Intramuscular	4 weeks	10 mg; 20 mg; 30 mg; 40 mg	30–72.2%	Hyperglycemia-related AEs (39.5–76.7%); diarrhea (39%); cholelithiasis (18.5–33%)
Roscovitine	Oral	Twice daily	400 mg	NA	NA
**ADRENAL-DIRECTED AGENTS**					
Metyrapone	Oral	Four/six times per day (one-three tablets per assumption)	250 mg	45.5–100%^§^	Hirsutism (36.1%, only women); dizziness (30.4%); arthralgias (13.4%)^§^
Osilodrostat	Oral	Once or twice daily	1 mg; 2 mg; 5 mg; 10 mg	66.4–91.7%	AI and hypocortisolism-related AEs (31.6–51.5%); fatigue or asthenia (28.5–58.3%); nausea (31.6–41.7%)
Levoketoconazole	Oral	Twice daily (one-four tablets per assumption)	150 mg	30.8–36.1%	Nausea (32%); headache (28%); peripheral oedema (19%)
ATR-101	Oral	Twice daily	125 mg; 250 mg; 500 mg	NA	NA
**GLUCOCORTICOID-RECEPTOR ANTAGONISTS**					
Mifepristone	Oral	Once daily (one-three tablets per assumption)	300 mg	38.1–60%	Nausea (48%); fatigue (48%); headache (44%)
Relacorilant	Oral	Once daily (one-four tablets per assumption)	50 mg; 100 mg	41.7–63.6%^#^	Back pain (31.4%); headache (25.7%); peripheral oedema (25.7%)^#^

*LAR, long-acting release; AE, adverse event; NA, not available*.

* Considered as UC or serum cortisol normalization for pituitary- and adrenal-directed agents, as glucose metabolism impairment and hypertension improvement for glucocorticoid-receptor antagonists

§ reported according to published studies, as the clinical trial is still ongoing without preliminary evidences

# *reported according to poster presentations*.

## Pituitary-Directed Drugs

Pituitary-directed drugs directly target the corticotroph pituitary tumor, which represents the origin of the disease and the source of the excessive ACTH production causing the adrenal cortisol excess, and consequently aim at the normalization of cortisol secretion through the control of the excessive ACTH secretion and the shrinkage of the pituitary tumor (4). The currently available pituitary-directed drugs include the somatostatin (SST) analogue (SSA) pasireotide and the dopamine agonist (DA) cabergoline; different formulations of pasireotide have been recently investigated in international, multicenter clinical trials, whereas cabergoline has been mainly experimented in single-center clinical experiences, although an international, multicenter clinical trial has been performed on the combination therapy of cabergoline with pasireotide. Although cabergoline is not currently under investigation in clinical trials, a brief summary of the available evidences will be provided. An additional pituitary-directed experimental drug, roscovitine, is under investigation in current clinical trials (4, 5).

Table 2 shows the characteristics of current and recent clinical trials, registered in clinicaltrials.gov, on pituitary-directed drugs for the treatment of CD in terms of official study name, identification code, study design, patient number, and start and completion date.

**Table 2:** Characteristics of current and recent clinical trials, registered in clinicaltrials.gov, on pituitary-directed drugs for the treatment of Cushing's disease in terms of official study name, identification code, study design, patient number, start, and completion date.

**Investigation drug**	**Official study name**	**ClinicalTrials.gov code**	**Study type**	**Estimated patient number**	**Start date**	**Completion date**
Pasireotide	A Multicenter, Open Label Study to assess the safety and efficacy of 600 μg SOM230, administered subcutaneously, b.i.d. in patients with Cushing's disease	NCT00088608	OL; P; MC; Phase II	39*	April 2004	June 2006
	Extension Study to assess the safety and efficacy of pasireotide in patients with Cushing's disease	NCT00171951	OL; P; MC; Phase II	19*	August 2004	July 2013
	A Randomized, Double-blind Study to assess the safety and efficacy of different dose levels of pasireotide (SOM230) subcutaneous (sc) over a 6 month treatment period in patients with *de novo*, persistent or recurrent Cushing's disease	NCT00434148	R; DB; P; MC; Phase III	162*	December 2006	May 2014
	An Open-label, Multi-center, Expanded Access Study of pasireotide s.c. in patients with Cushing's disease. (SEASCAPE)	NCT01582061	OL; P; MC; Phase III	104*	August 2011	January 2017
	Non-interventional Study for the generation of long term safety and efficacy data of pasireotide s.c. in patients with Cushing's disease (post-authorization safety study)	NCT02310269	OL; P; MC; Phase IV	200	March 2013	May 2024
Pasireotide LAR	A Randomized, Double-blind, Multicenter, Phase III Study to evaluate the efficacy and safety of pasireotide LAR in patients with Cushing's disease	NCT01374906	R; DB; P; MC; Phase III	150*	November 2011	December 2016
Roscovitine	Treatment of pituitary Cushing disease with a selective CDK inhibitor, R-roscovitine	NCT02160730	OL; P; SC; Phase II	7*	May 2014	October 2018
	A Phase 2 Multicenter Study of Seliciclib (R-roscovitine) for Cushing disease	NCT03774446	OL; P; MC; Phase II	29	November 2018	November 2022

*b.i.d., bis in die; OL, open-label; P, prospective; MC, multicenter; R, randomized; DB, double-blinded; SC, single-center*.

* *Definitive patient number due to study completion*.

### Pasireotide

Pasireotide is a novel subcutaneously injective multi-receptor ligand SSA, and it is currently the only pituitary-directed drug with an official license by the European Medical Agency (EMA) in April 2012 and the American Food and Drug Administration (FDA) in December 2014 for the treatment of CD in patients for whom surgery is not an option or for whom surgery has failed or has not been curative.

SST is a hypothalamic modulator of the pituitary function, mainly involved in growth hormone secretion (6). Some experimental studies have suggested a possible SST role in the inhibition of pituitary ACTH secretion, through the activation of SST receptor (SSTR) type 2 (SSTR2) and type 5 (SSTR5), expressed on the pituitary corticotroph cells (7). However, the administration of octreotide, an SSA with high affinity for SSTR2, failed to show any significant decrease in circulating ACTH and, consequently, cortisol levels, both in healthy subjects and in CD patients (8, 9). More recently, experimental studies on human ACTH-secreting tumor cell cultures have shown that, after incubation with dexamethasone, SSTR2 expression significantly decreased, with SSTR5 expression remaining stable (7, 10), suggesting that chronic glucocorticoid exposure is able to downregulate SSTR2, but not SSTR5, expression, which could be consequentially considered as the real potential target for SSA treatment in CD (4, 7). On the basis of this evidence, in the early 2000s, preclinical and clinical investigations were started on a multi-ligand SSA, pasireotide, with relevant affinity for SSTR type 1 (SSTR1), SSTR2, SSTR type 3 (SSTR3), and particularly for SSTR5 (11). Noteworthy, compared to octreotide, pasireotide has a 30-, 11-, and 158-fold higher affinity to SSTR1, SSTR3, and SSTR5, respectively, as well as a 7-fold lower affinity to SSTR2 (4), therefore suggesting a differential use of pasireotide compared with conventional SSAs. Experimental studies on murine corticotroph pituitary tumor cell lines and human corticotroph pituitary tumor cell cultures demonstrated that pasireotide induced a significant decrease in ACTH secretion (4, 7); interestingly, the inhibition of ACTH secretion was maintained even in cultures pre-incubated with dexamethasone, supporting the hypothesis of a predominant role of SSTR5 in the regulation of corticotroph cell secretion during chronic hypercortisolism (7, 10). This evidence was also confirmed in animal models, since pasireotide treatment induced a significant decrease in ACTH and corticosterone levels in rats (12). In subsequent phase I studies on healthy subjects, pasireotide showed a half-life of about 7–11 h after a single administration at doses ranging from 2.5 to 1,200 μg (13), allowing a twice-daily administration, and a good safety profile, mainly associated with gastrointestinal AEs (13–16). Pasireotide, as subcutaneous injection, is formulated as three different immediate-release ampoules, containing 300, 600, and 900 μg doses, with suggestion of a maximum total daily dose of 1,800 μg.

Pasireotide has been extensively evaluated in human clinical studies, leading to a large amount of evidence about its efficacy and safety in CD treatment (17–30). The first classical formulation of pasireotide was subcutaneously administered in a twice-daily schedule, with two doses, 600 and 900 μg. Pasireotide treatment was originally investigated in a phase II, proof-of-concept, open-label, single-arm, multicenter clinical trial (clinicaltrials.gov code: NCT00088608) performed to assess the efficacy and safety in a limited population of patients with CD for a period of 12 weeks. In this study, 39 CD patients with mean urinary cortisol (mUC) ≥2-fold the upper limit of normal (ULN) were enrolled and entered a 2-week, stable-dose period. Pasireotide was started and maintained at a dose of 1,200 μg/day, unless a dose down-titration was required due to safety issues. The primary endpoint of this study was the rate of patients with mUC normalization, defined as mUC within the normal ranges, after 2 weeks of treatment. After 2 weeks of treatment, pasireotide induced a decrease of mUC in 22 (75.9%) of the 29 patients included in the efficacy analysis, with 11 (37.9%) patients displaying a >50% mUC decrease and five (17.2%) patients reaching mUC normalization. Moreover, a decrease in plasma ACTH and serum cortisol levels was observed. The most frequently reported AEs were diarrhea (43.6%), hyperglycemia (35.9%), and nausea (23.1%), whereas three (7.7%) patients experienced serious AEs, consisting of hyperglycemia in two (5.1%) patients and a cardiac event in one (2.6%) patient (17). Treatment discontinuation occurred only in one (2.6%) patient, because of hyperglycemia (17).

The main clinical trial on pasireotide treatment was a phase III, randomized, double-blind, multicenter clinical trial (clinicaltrials.gov code: NCT00434148), performed to assess the efficacy and safety in a wide population of CD patients for a period of 12 months. In this study, 162 CD patients with mUC ≥1.5-fold the ULN were enrolled and were double-blindly randomized in two cohorts, according to the assigned starting dose of 1,200 μg/day (82 patients) or 1,800 μg/day (80 patients) (18). The two cohorts of patients entered a 3-month, double-blind period. At month 3, patients with mUC ≤2-fold the ULN and not exceeding the baseline levels continued to receive their randomly assigned dose in a double-blind period for three additional months; the remaining patients were unblinded and received an additional 600 μg daily for 3 months with a dose up-titration to 1,800 or 2,400 μg/day, according to the assigned cohort. The primary endpoint of this study was the rate of patients with normalization of mUC, defined as mUC ≤ ULN, or full control, at month 6 without a prior dose up-titration. After 6 months of treatment, pasireotide induced mUC normalization in 33 (20.4%) patients without dose up-titration and in 36 (22.2%) patients regardless of dose up-titration. Moreover, a ≥50% mUC decrease, or partial control, was achieved in additional 25 (15.4%) patients regardless of dose up-titration. Therefore, a total success rate, including normalization and a ≥50% mUC decrease regardless of dose change during the study, was registered in 61 (37.6%) patients. After 12 months of treatment, pasireotide induced mUC normalization in 31 (19.1%) and a ≥50% mUC decrease in 15 (9.3%) patients regardless of dose up-titration, with total success rate in 46 (28.4%) patients. Noteworthy, patients treated with 1,800 μg/day had a higher rate of mUC normalization regardless of dose up-titration compared with patients treated with 1,200 μg/day of pasireotide, both at month 6 (29 vs. 16%) and 12 (25 vs. 13%). Considering the overall population, mUC rapidly decreased during the first 3 months of treatment, with a median decrease of 50% that remained stable throughout the study (18). Among the 72 patients with uncontrolled hypercortisolism, defined as mUC > ULN and a <50% mUC decrease compared to baseline levels, at months 1 and 2, hypercortisolism remained uncontrolled in 66 (92%) patients at month 6 and in 64 (89%) patients at month 12, suggesting that the absence of response to pasireotide may be predicted in the first months of treatment (4, 18). A decrease in mean plasma ACTH, serum cortisol, and late-night salivary cortisol (LNSC) levels was observed at month 12 (18). Regarding clinical profile, after 12 months of treatment, a significant decrease in body weight, waist circumference, and total cholesterol was observed in fully and partially controlled, as well as uncontrolled patients, whereas a significant decrease in low-density lipoprotein (LDL) cholesterol was reported in fully controlled and uncontrolled patients (18, 19). In particular, in patients with dyslipidemia at baseline, a decrease in total and LDL-cholesterol levels was observed, regardless of whether patients were receiving lipid-modifying medication during the study (19). Despite a non-significant decrease in the total population of patients, a significant decrease in blood pressure was observed in hypertensive patients regardless of whether patients were receiving antihypertensive medication during the study (19). Improvement in quality of life was also observed in fully and partially controlled patients (18–20), with the highest improvement reached by fully controlled patients (20), whereas depressive status improved in fully and partially controlled, as well as uncontrolled, patients (19). The evaluation of pasireotide effect on pituitary tumor was firstly reported on the 75 (46%) patients with a measurable tumor at baseline on magnetic resonance imaging (MRI). Tumor shrinkage was observed, both at month 6 and 12, with a mean tumor volume decrease of 5.7% (mean tumor volume increase of 9.3% in the 1,200 μg group and tumor volume decrease of 19% in the 1,800 μg group) and of 28.6% (mean tumor volume decrease of 9.1% in the 1,200 μg group and 43.8% in the 1,800 μg group) (18, 27), respectively. In particular, according to a recent *post-hoc* analysis, considering the 53 patients with measurable tumor volume at both baseline and month 6, a tumor shrinkage ≥25% was observed in 20 (37.7%) patients, whereas, considering the 32 patients with measurable tumor volume at both baseline and month 12, a tumor shrinkage ≥25% was observed in 18 (56.2%) patients (27). Noteworthy, both at month 6 and 12, tumor volume reduction was more frequently observed in patients treated with 1,800 μg/day than in patients treated with 1,200 μg/day (27). In a report on eight patients, followed-up in one of the main centers participating at the clinical trial, a tumor shrinkage >25% was observed in five (62.5%) patients after 6 months and in the totality (100%) of patients after 12 months, with a mean tumor shrinkage of 42.4 and 61.6%, respectively, and tumor mass disappearance in one (14.3%) case after 12 months of treatment (23). Regarding safety profile, the most frequently reported AEs were diarrhea (58%), nausea (51.8%), and hyperglycemia (40%), whereas serious AEs were reported in 40 (24.7%) patients, mainly represented by diabetes mellitus (3.1%), hyperglycemia (2.5%), cholelithiasis (2.5%), and adrenal insufficiency (AI) (1.2%) (18, 28). AEs were also grouped in categories of special interest, including hyperglycemia-related AEs (73%), gallbladder-related AEs (30%), liver safety-related AEs (29%), hypocortisolism-related AEs (8%), and QT prolongation (1.8%). Treatment discontinuation occurred in 84 (51.8%) patients, because of AEs in 26 (16%) and lack of efficacy in 37 (22.8%) patients (18).

Following the first two main clinical trials, additional clinical studies focused on long-term response to pasireotide treatment (21–25, 28–30). In an extension study to 24 months of the phase II trial (clinicaltrials.gov code: NCT00171951) on 19 CD patients, considering the 18 patients included in the primary efficacy analysis, pasireotide treatment at doses ranging from 300 to 1,800 μg/day demonstrated mUC normalization in four (22.2%) patients and a reduction in mUC, defined as lower than that at core study baseline, but not within normal range, in additional six (33.3%) patients after 6 months of treatment, with a total success rate of 55.5%. Moreover, considering the four patients who reached the 24 months follow-up, one (25%) normalized mUC and three (75%) had a >50% mUC decrease from core study baseline. A decrease in body weight and diastolic blood pressure was reported. The most frequently reported AEs, registered from the baseline of the core study to the end of the extension study, were diarrhea (68.4%), nausea (63.2%), and hyperglycemia (57.9%). Overall, hyperglycemia-related AEs occurred in 13 (68.4%) patients. Treatment discontinuation during the extension phase occurred in 16 (84.2%) patients, because of AEs in one (5.3%) and lack of efficacy in three (15.8%) patients (21).

In an extension study to 24 months of the main phase III trial on 58 CD patients, including 29 (50%) with mUC normalization and 12 (20.7%) with a ≥50% mUC decrease from core baseline at the end of the core study, pasireotide treatment with doses ranging from 600 to 2,400 μg/day demonstrated mUC normalization in 20 (34.5%) patients and a ≥50% mUC decrease in additional five (8.6%) patients, with total success rate of 43.1%, after 24 months of treatment. Interestingly, 14 (48.3%) of 29 patients with mUC normalization at 12 months in the core study remained with mUC within normal values at 24 months. A decrease in body weight, blood pressure, and total and LDL cholesterol was reported, with a significant improvement after 12 months and a further improvement after 24 months of treatment (22). The most frequently reported AEs, registered from the baseline of the core study to the end of the extension study, were diarrhea (55.6%), nausea (48.1%), and hyperglycemia (38.9%) (22). Treatment discontinuation during the extension phase occurred in 27 (46.6%) patients, because of AEs in five (8.6%) and lack of efficacy in 10 (17.2%) patients.

In another extension study to 60 months of the main phase III trial on 16 CD patients, treated with doses ranging from 300 to 2,400 μg/day, pasireotide induced mUC normalization in 11 (68.8%) patients and a ≥50% mUC decrease from core baseline in additional two (12.5%) patients after 60 months of continuous treatment, with a median percentage decrease in mUC of 82.6% after 12 months and 81.8% after 60 months. Interestingly, eight (72.7%) of 11 patients with mUC normalization at 60 months were stably controlled from month 12 of treatment (25). A decrease in body weight and blood pressure was observed, being already present at 6 months and persisting until the 60 months of treatment (25). The most frequently AEs, reported from the baseline of the core study until the end of the extension study, were nausea (68.8%), hyperglycemia (56.3%), cholelithiasis (50%), abdominal pain (50%), diabetes mellitus (50%), fatigue (50%), and myalgia (50%). Noteworthy, hyperglycemia-related AEs were reported in 93.8%, gallbladder/biliary-related AEs in 62.5%, bradycardia-related AEs in 25%, and liver-safety-related AEs in 18.8% of patients (25).

In an Italian study on 20 CD patients, 12 initially enrolled in the phase II (six patients) and III studies (six patients) and the remaining eight treated with pasireotide after commercialization, pasireotide treatment at a mean dose of 1,320–1,410 μg/day (range: 600–2,400 μg/day) induced mUC normalization in 10 (50%) patients, after a mean treatment duration of 20.5 months (24). A sustained decrease in body weight, waist circumference, and blood pressure was observed (24). The most frequently reported AEs included gastrointestinal disturbances, comprising diarrhea, abdominal pain, and decreased appetite, together with newly-onset diabetes or worsening of previous glucose metabolism impairment (85%), cholelithiasis in patients with baseline normal gallbladder at abdomen ultrasounds (33%), AI (10%), and liver enzyme elevation (10%) (24).

Recently, a multicenter, real-world evidence, Italian study on 32 CD patients demonstrated a relevant efficacy of pasireotide treatment. According to an “intention-to-treat” approach, including the 31 patients with very mild to moderate disease, with the exclusion of a single patient with very severe disease, UC normalization (UC ≤ ULN) or near-normalization (UC >1 and ≤ 1.1 ULN) was reported in 19 (61.3%) and in two (6.4%), respectively, of the 31 patients with very mild to moderate CD after 6 months of treatment, at doses ranging from 600 to 1,800 μg/day, demonstrating a total success rate of 67.7% (26). Conversely, according to a “per-protocol” approach, including only the 26 patients with very mild to moderate disease reaching the 6 months follow-up, UC normalization or near-normalization was reported in 19 (73.1%) and in two (7.7%) patients, respectively, demonstrating a total success rate of 80.8% (26). A decrease in body weight, waist circumference, and total and LDL cholesterol was observed (26). The most frequently reported AEs were hyperglycemia (81.2%), diarrhea (37.5%), and asthenia (18.7%) (26). The reason behind the apparent discrepancy on the success rate between clinical trials and the real-world evidence study mainly relies on the strict protocol requirements in terms of inclusion and exclusion criteria, study design, and efficacy and safety analyses adopted in the phase III study. Indeed, in the phase III study, patients achieving mUC normalization through pasireotide up-titration were considered as non-responders (18), whereas in clinical practice UC normalization is considered the main endpoint, regardless of dose adjustments required during the follow-up. Moreover, efficacy analysis in the phase III study was performed according to an “intention-to-treat” approach, classifying thereof as non-responders not only patients unable to reach mUC normalization at the end of the study, but also patients who discontinued pasireotide throughout the study, therefore unavoidably increasing the rate of treatment failure (18). This concept was clearly demonstrated in the real-world evidence study (26), where both “intention-to-treat” approach, which considers the totality of patients starting the study, with those who discontinued classified as non-responders, and “per-protocol” approach, which considers only patients who completed the study, were adopted in the efficacy analysis, reporting a success rate of 67.7 and 80.8%, respectively (26). Moreover, it should be noted that the real-world evidence study mainly enrolled patients with very mild (mUC <1.5 ULN), mild (mUC ≥1.5 and ≤ 2 ULN), and moderate (mUC >2 and ≤ 5 ULN) disease, where UC normalization could be more easily reachable (26), whereas the phase III study also enrolled patients with severe (mUC >5 and ≤ 10 ULN) and very severe (mUC >10 ULN) disease, representing more than one third of the total population, where mUC normalization could be less easily reachable (18). Therefore, it could be argued that the true pasireotide efficacy could have been underestimated in the phase III clinical trial.

Such consideration may be supported by the evidences reported in an *interim* analysis of an on-going multicenter, post-authorization, observational, real-world evidence study (clinicaltrials.gov code: NCT02310269), expecting to enroll 200 CD patients, where pasireotide treatment at a median average dose of 1,200 μg/day (range: 200–1,800 μg/day) induced mUC normalization in 27 (81.8%) of 33 and in 12 (63.2%) of 19 patients treated for 12 and 24 months, respectively, confirming the hypothesis of a higher pasireotide efficacy in the real-life setting (29). However, in an additional multicenter, post-authorization, observational study on real-life experience with pasireotide treatment (clinicaltrials.gov code: NCT01582061) in 104 CD patients, mUC normalization was reached in 36 (54.5%) of 66, 22 (47.8%) of 46, and nine (42.9%) of 21 patients treated with pasireotide at a mean dose of 1,421 μg/day for 12, 24, and 48 weeks (30), respectively, reporting thereof results similar to those of the phase III clinical trial (18). An improvement in body weight, waist circumference, and blood pressure was observed, as well as an improvement in quality of life. The most frequently reported AEs were diarrhea (51%), nausea (46.2%), and hyperglycemia (40.4%). Treatment discontinuation occurred in 64 (61.5%) patients, because of AEs in 20 (19.2%) and lack of efficacy in 26 (25%) patients (30). On the basis of these evidences, further real-life studies are required to draw definitive conclusions on the efficacy of pasireotide in the routine clinical setting.

The clinical trials on pasireotide treatment in its classical subcutaneous formulation displayed some pitfalls, mainly represented by the frequent occurrence of hyperglycemia, often requiring additional antidiabetic treatments, and by the administration schedule, requiring a twice-daily subcutaneous injection. Therefore, to reduce the burden of treatment, a new, long-acting release (LAR) pasireotide formulation, denominated pasireotide LAR, requiring a single, intramuscular administration every 4 weeks, has been developed in recent years. In a phase I study on healthy subjects, pasireotide LAR displayed a sustained release of pasireotide over 1 month and a good safety profile, mainly associated with gastrointestinal AEs (31). Pasireotide LAR is formulated as four different ampoules, containing 10, 20, 30, and 40 mg doses, with suggestion of a maximum total dose of 40 mg every 4 weeks.

A phase III, randomized, double-blind, multicenter clinical trial (clinicaltrials.gov code: NCT01374906) was started to assess efficacy and safety of pasireotide LAR treatment on CD patients for a period of 12 months (32). Pasireotide LAR was intramuscularly administered in a once-monthly schedule. In this study, 150 CD patients, displaying baseline mUC between 1.5- and 5-fold the ULN, were enrolled and were double-blindly randomized in two cohorts, according to the assigned starting dose of 10 mg (74 patients) or 30 mg (76 patients) every 4 weeks. The two cohorts entered a 4-month, double-blind, stable-dose period. At month 4, patients with mUC ≤ 1.5-fold the ULN were maintained at stable dose, whereas in the remaining patients, dose up-titration from 10 to 30 mg and from 30 to 40 mg, respectively, was performed. The whole cohort of patients continued the double-blind period until month 7, when they entered a final 5-month, single-blind period, during which patients with mUC normalization (mUC ≤ ULN) were maintained at stable dose, whereas in the remaining patients, dose up-titration was performed at month 7, 9, or 12, till a maximum dose of 40 mg (32). The primary endpoint of this study was the proportion of patients achieving a mUC normalization at month 7, regardless of dose up-titration at month 4, in each randomized dose group. After 7 months of treatment, pasireotide LAR induced mUC normalization in 62 (41.3%) patients regardless of dose up-titration, with 45 (30%) patients reaching mUC normalization without dose up-titration. Moreover, after 12 months of treatment mUC normalization was observed in 45 (30%) patients regardless of dose up-titration. Median mUC decreased in the first 4 weeks of treatment in both groups and stably remained below the ULN throughout the study. A decrease in plasma ACTH and LNSC levels was observed in both cohorts at month 12, and changes in serum cortisol concentrations were generally consistent with plasma ACTH and LNSC concentrations (32). Regarding clinical profile, after 12 months of treatment, a decrease in body weight, waist circumference, and blood pressure was observed, together with an improvement in quality of life (32, 33). Moreover, in patients with evaluable measurements at pituitary MRI at both baseline and month 12, a median tumor shrinkage of 17.8% was observed in the 35 patients of 10 mg group and of 16.3% in the 38 patients of 30 mg group; in particular, a decrease in tumor volume ≥20% compared with baseline volume occurred in 15 (43%) of the 35 patients of 10 mg group and in 18 (47%) of the 38 patients of 30 mg group (32). Regarding safety profile, the most frequently reported AEs were hyperglycemia (48%), diarrhea (39%), and cholelithiasis (33%), whereas serious AEs were reported in 38 (25.3%) patients, mainly represented by cholelithiasis (2.7%). AEs were also grouped in categories of special interest, including hyperglycemia-related AEs (76.7%), gallbladder or biliary-related AEs (34.7%), liver safety-related AEs (20%), injection site-related AEs (2.7%), and QT prolongation (1.3%). Treatment discontinuation occurred in 46 (30.7%) patients, because of AEs in 13 (8.7%) and lack of efficacy in 18 (12%) patients (32). Some slight differences in hormonal control were observed in the two cohorts after 12 months of treatment, as 26 (35%) patients of the 10 mg group normalized mUC, compared with 19 (25%) patients of the 30 mg group, showing slightly higher efficacy in the lower dose group (32). The reasons behind these surprising results are unclear; it could be argued that patients of the 30 mg group, compared to patients of the 10 mg group, included a higher percentage of macroadenomas (38 vs. 27%) (32), potentially suggesting a more aggressive tumor behavior and thus explaining the difference in terms of hormonal control. Moreover, it should be noted that the rate of controlled patients of the 10 mg group may have been influenced by the up-titration occurred at month 4, when uncontrolled patients of the 10 mg group had a dose up-titration to 30 mg and uncontrolled patients of the 30 mg group had a dose up-titration to 40 mg, respectively (32); indeed, the number of controlled patients of the 30 mg group was significantly higher before month 4 compared with the number of controlled patients of the 10 mg group, whereas thereafter similar rates were observed (32). However, further studies are required to ultimately clarify the effect of different doses of pasireotide LAR on cortisol control in patients with CD.

In a subsequent, extension study to 36 months of the phase III clinical trial, 81 CD patients were treated with pasireotide LAR for a median follow-up of 23.9 months (34). After 24 months of treatment at doses ranging from 5 to 40 mg every 4 weeks, pasireotide LAR induced mUC normalization in 38 (46.9%) patients, with 12 (14.8%) patients requiring a dose up-titration during the 12 months of extension after the core study. Noteworthy, 27 (64.3%) of 42 patients with controlled mUC at 12 months, at the end of the core study and starting of extension, maintained mUC normalization at 24 months. Moreover, considering a subgroup of 18 patients reaching 36 months of continuative treatment, mUC normalization was reached in 13 (72.2%) patients (34). A decrease in body weight, waist circumference, and blood pressure was sustained during the extension, together with the improvement in quality of life. Of the 35 patients with a measurable tumor at MRI of core baseline and month 24, tumor shrinkage ≥20% was observed in 12 (34.3%) patients. The most frequently reported AEs were hyperglycemia (23.5%), nasopharyngitis (19.8%), and cholelithiasis (18.5%) (34). Hyperglycemia-related AEs occurred in 32 (39.5%) patients (34). Treatment discontinuation occurred in 42 (51.8%) patients, because of AEs in eight (9.9%) and lack of efficacy in 12 (14.8%) patients (34). On the basis of this evidence, pasireotide LAR has been officially licensed by EMA in July 2017 and by FDA in June 2018 for the treatment of CD in patients for whom surgery is not an option or for whom surgery has failded or has not been curative.

Overall considered, the classical short-acting subcutaneous formulation of pasireotide represents an effective treatment for CD, particularly in patients with mild to moderate disease, in whom pasireotide treatment may be considered an interesting approach. The long-lasting improvement in clinical profile and the potential tumor shrinkage suggest to consider long-term treatment in responsive patients, with accurate dose adjustments, according to the disease control and safety, and to prefer the treatment in patients with visible and particularly large or invasive pituitary tumors, with consequent potential help for a radical surgical resection in case of subsequent pituitary surgery. However, the frequent occurrence of hyperglycemia induces to consider the treatment with subcutaneous pasireotide preferable in patients without severe or uncontrolled diabetes and suggests a strict monitoring of glucose profile in patients with baseline impairment of glucose metabolism. Nevertheless, the twice-daily subcutaneous injection might be not always comfortable, with potentially discouragement of patients from properly following treatment schedule, and a potential negative impact on success rate. On the other hand, the novel long-acting intramuscular formulation of pasireotide showed similar or slightly superior efficacy compared to the subcutaneous formulation, in both short-term and long-term treatments, sharing a similar safety profile, and therefore may be considered in CD patients eligible for pasireotide treatment with the potential advantage to improve compliance and probably success rate, nevertheless associated with a strict monitoring of glucose profile, especially in patients with baseline impairment of glucose metabolism or diabetes.

### Roscovitine (Seliciclib)

Roscovitine, or seliciclib, is an oral anticancer agent, acting as an inhibitor of the cyclin-dependent kinases (CDKs) family, that has been under clinical investigation for different types of malignancies during the last 20 years (35).

CDKs allow a normal cell cycle progression through the regulation of cell cycle checkpoints, for which they need to bind specific regulatory subunits, called cyclins, creating therefore active CDK/cyclin regulatory complexes (36). CDK/cyclin complexes are differentially expressed during the cell cycle, with specific CDK/cyclin complexes involved for each cell cycle phase, on different cell pathways. Therefore, a gradual hierarchical activation of CDK/cyclin complexes is required for each cell cycle phase to proceed to the subsequent phase (36). Overall, CDK/cyclin complexes activity prevents instable or excessive cell proliferation, and their alterations, mainly characterized by cyclin overexpression and subsequent CDK/cyclin complexes overactivation, may lead to an abnormal increase in cell proliferation and survival (36–38). Therefore, CDK/cyclin complexes have been considered as potential targets for new anticancer drugs; indeed, clinical research has focused on compounds targeting CDKs, developing both single-target and multi-target kinase inhibitors (35). Among these compounds, roscovitine has shown promising experimental results, being able to inhibit many CDK/cyclin complexes, including CDK1/cyclin B, CDK2/cyclin A, CDK2/cyclin E, CDK5/p35, CDK7/cyclin H, and CDK9/cyclin H (35). Further experimental evidences on neoplastic cell lines, including L1210 mouse leukemia, A549 and MR65 human lung cancer, and CHP212 human neuroblastoma cell lines, demonstrated that roscovitine inhibited cell proliferation and promoted cell apoptosis (35). Moreover, several experimental evidences reported a synergistic effect in the control of cell proliferation and in the induction of autophagy when roscovitine was used in combination with chemotherapeutic agents (35). However, studies in xenograft mice injected with different human neoplastic cell lines, including MES-SA/Dx5 uterine sarcoma, LOVO colorectal adenocarcinoma, and A4573 Ewing sarcoma-derived cell lines, demonstrated mild effects of roscovitine as a single anticancer treatment, since it induced a delay in tumor growth rather than a shrinkage of tumor mass (35). Conversely, additional studies in mice injected with different human primary cultures, including Epstein-Barr Virus (EBV)-positive human primary cultures from nasopharyngeal cancer tumors (39), or cell lines, including H358 non-small cell lung cancer (40) and GBM43 glioma (41), testing roscovitine in combination with additional anticancer treatments, including radiation therapy (39), erlotinib (40) or phosphoinositide-3 (PI-3) kinase inhibitors (41), demonstrated that the combination treatments reduced the initial tumor volume of a degree up to 75%, with no specific combined treatment showing a clear superiority in terms of tumor volume decrease or stabilization (39–41). In humans, roscovitine, as a single anticancer treatment, showed a response on tumor growth in about half of patients, mainly inducing disease stabilization, in phase I (42, 43) and phase II (35) clinical studies on patients with solid tumors and non-small cell lung cancer, respectively.

Recently, roscovitine has been suggested as a potential treatment for CD, based on the evidence of neoplastic pituitary origin of CD with potential role for CDK/cyclin complexes in pituitary tumorigenesis. Indeed, *in vitro* and *in vivo* studies have shown cyclin E overexpression in corticotroph pituitary tumors, mainly associated with dysregulation in *pituitary tumor transforming gene* (*PTTG*) and in *Brahma-related gene-1 (Brg1)* genes, both involved in the regulation of corticotroph cells cycle (44–46). Indeed, *PTTG* overexpression and *Brg1* loss of function may lead to an increase in cyclin E intracellular levels, favoring corticotroph cells proliferation (44–46). Therefore, two experimental studies were performed to evaluate the potential role of roscovitine in the treatment of CD (46, 47). The first study was mainly focused on the effect of roscovitine in animal models, particularly in zebrafish transgenic and murine xenograft models of pituitary corticotroph tumor, with additional report of *in vitro* evidences on AtT20, the mouse corticotroph tumor cell line (46). The second study reported the effect of roscovitine in human corticotroph pituitary primary cell cultures (47). In the first study, in a zebrafish model, *PTTG* gene overexpression was induced by the transposition to a full-length *PTTG* gene of a proximal promoter of *proopiomelanocortin (POMC)* gene, which regulates the expression of pro-opiomelanocortin (POMC), the ACTH precursor, and it resulted in dysregulated and uncontrolled cell proliferation in two (33.3%) of six cases, leading to a neoplastic lesion, strongly resembling the human corticotroph tumor, and representing a model of CD. Interestingly, the mutant zebrafish embryos were exposed to different CDK inhibitors, including roscovitine, which reduced POMC expression of about 40% compared with controls. Moreover, *in vitro* evaluation on AtT20 mouse corticotroph tumor cell line demonstrated that roscovitine decreased ACTH secretion and cell proliferation, inhibiting cyclin E and inducing cell cycle inhibitors (p27^Kip1^, p57^Kip2^, and p21^Cip1^) expression, as well as inhibiting phosphorylation of retinoblastoma protein (46). In the *in vivo* evaluation in athymic nude mice injected with AtT20 corticotroph tumor cell line, with consequent development of local subcutaneous corticotroph tumors, representing a model of CD, roscovitine induced a reduction of tumor mass of about 50% compared with controls after 3 weeks of treatment, together with a decrease in plasma ACTH and serum corticosterone levels, which were significantly lower in mice treated with roscovitine than in controls (46). In the second study, cell cultures of surgically-resected human corticotroph pituitary tumors were treated with roscovitine, which induced an up to 62% decrease in ACTH secretion in five (83.3%) of six different cell cultures (47). Moreover, in three tumor cell cultures, a 30–50% decrease in POMC messenger expression was identified, probably related to POMC promoter and transcriptional factors inhibition (47). These preliminary data suggested that roscovitine treatment is potentially useful in the control of ACTH secretion and pituitary tumor growth.

Presently, no evidences on roscovitine treatment in CD patients are available in humans. A first phase II, proof-of-concept, open-label, single-center clinical trial (clinicaltrials.gov code: NCT02160730), evaluating the efficacy of roscovitine in patients with CD for a period of 4 weeks, started in May 2014 and was completed in October 2018. The study enrolled seven CD patients, treated with roscovitine at the dose of 800 mg/day, divided in a twice-daily administration for 4 days a week, along 4 weeks of treatment. The primary endpoint of this study was the prevalence of normalization of UC after 4 weeks of roscovitine treatment. However, no results of the trial are currently available. A second phase II, open-label, multicenter clinical trial (clinicaltrials.gov code: NCT03774446), evaluating the efficacy of roscovitine in patients with CD for a period of 4 weeks, started in November 2018 and is currently on-going. The study expects to enroll 29 CD patients, to treat with roscovitine up to 800 mg/day for 4 days a week, along 4 weeks of treatment. The primary endpoint of this study was the number of participants with a normalized UC and with UC above the ULN, but reduced by ≥50% from baseline at study completion. The expected study completion date is November 2022, but no preliminary results are currently available. Therefore, nowadays, it is not possible to draw conclusions on the potential future employment of roscovitine in the clinical setting of the management of CD.

### Cabergoline

Cabergoline is an oral DA, acting on the entire cohort of the five dopamine receptors (DR), belonging to the stimulatory D1-like receptors, which include D1 and D5 receptor subtypes, and the inhibitory D2-like receptors, including D2, D3, and D4 receptors subtypes, although the highest affinity is shown for D2-like receptors, especially D2 receptors. Cabergoline is currently suggested as a second-line, off-label treatment for CD (4).

D2 receptors are widely expressed in the anterior and intermediate lobes of the pituitary gland, where they are mainly involved in the tonic regulation of prolactin (PRL) and melanocyte stimulating hormone (MSH) secretion by lactotroph and melanotroph cells, respectively, mediating the inhibitory effect of hypothalamic dopaminergic pathways. However, it has been shown that D2 receptors are also expressed in different pituitary cell populations, and that a relevant D2 expression may be found in different types of pituitary tumors, including corticotroph pituitary tumors (4, 48, 49). Therefore, DAs, particularly cabergoline, have been used as medical therapy for the management of various pituitary tumors, and have been suggested as potential medical treatment for CD (4). Cabergoline has shown a long half-life, ranging 63–109 h (50), allowing therefore a potential once-weekly administration at the dose of 0.5 mg, although different administration schedules, from once-weekly to once-daily administrations, have been tested in different diseases (4, 51). Cabergoline is formulated as an immediate-release tablet, containing a 0.5 mg dose.

Although case reports on the use of cabergoline in CD treatment have been published since the early 2000s, the first prospective, single-center, open-label clinical study on cabergoline treatment in 20 patients with persistent CD after pituitary surgery, with mUC ≥1.5-fold the ULN, was performed at the end of decade (52). After 3 months of treatment, cabergoline with doses ranging from 1 to 7 mg/week induced mUC normalization, defined as mUC ≤ ULN, in seven (35%) patients, and a ≥25% mUC decrease in additional eight (40%) patients, with a total success rate of 75%; five (33.3%) of these 15 patients experienced a treatment escape after 12–18 months (52). After 24 months of treatment, with a median dose of 3.5 mg/week, mUC normalization was reported in eight (40%) patients (52). Moreover, improvement in the clinical syndrome, as well as in the comorbidities, mainly hypertension and impairment of glucose metabolism, was observed, and tumor shrinkage was registered in four (50%) of the eight patients responsive at month 24 (52). Furthermore, cabergoline showed to be a safe treatment, as the most frequently reported AEs were asthenia (30%), hypotension (10%), and dizziness (5%). Noteworthy, a pre-existing mild tricuspid regurgitation progressed to a moderate degree, with normal pulmonary pressure, at the end of study in one (5%) patient, whereas no other newly onset or worsening of pre-existing cardiac valve insufficiency was observed (52).

After this initial clinical study (52), six additional clinical studies were published on the use of cabergoline as a treatment for CD (53–58), five of which (53, 55–58) have recently been reviewed, together with the initial Italian study (52), in a meta-analysis on the use of cabergoline in monotherapy in CD patients, including studies using UC normalization as a primary endpoint (59). According to this meta-analysis, cabergoline monotherapy induced UC normalization in 39.4% of patients, with a median UC decrease of 32.2%, at median doses of 3 mg/week (range: 0.5–7 mg/week) (59). Noteworthy, the meta-analysis showed that responder patients had baseline UC significantly lower than non-responder patients (59), suggesting a higher chance of success in patients with mild compared to patients with severe disease. Among the 42 patients who responded to cabergoline monotherapy, data for long-term treatment response were available for 36 patients, out of which eight (22.2%) experienced treatment escape (59). Particularly, in three studies analyzing long-term cabergoline monotherapy, treatment escape was reported at a rate variable from 18.2 to 38.9% of the initially responsive patients (52, 53, 58). The meta-analysis reported mild to moderate AEs in 37.3% of patients, mainly represented by nausea (13.5%), asthenia (11%), and vertigo (3.2%) (59). Noteworthy, considering the four studies in which echocardiographic evaluations were performed (52, 53, 55, 56), the previously reported worsening in tricuspid regurgitation was the only reported AE related to cardiac valve insufficiency in patients treated with cabergoline (52, 59).

In an Indian study conducted on 20 persistent or recurrent CD patients (54), using normalization of midnight serum cortisol and serum cortisol response to low-dose dexamethasone suppression test as primary endpoints, and therefore not included in the meta-analysis (59), cabergoline monotherapy normalized midnight serum cortisol or cortisol response to dexamethasone in seven (35%) patients after 12 months of treatment; however, five (71.4%) responder patients had previously received pituitary radiotherapy and two (28.6%) responder patients remained controlled even after cabergoline withdrawal, thus suggesting a possible effect on disease control by radiotherapy; therefore, excluding the previously reported two patients, a response to cabergoline treatment can be attributed to five (27.8%) of 18 patients, showing a success rate slightly lower than 30% (54).

Regarding clinical profile, whose evaluation was beyond the scope of the meta-analysis (59), in the available studies, cabergoline treatment improved clinical syndrome and comorbidities, particularly hypertension and glucose metabolism impairment (52–56, 58), whereas, with the exception of the previously reported Italian study (52), only two studies properly reported pituitary tumor evaluation at baseline and after cabergoline treatment, not showing any significant change in tumor volume (54, 56).

Despite the growing body of evidences reported in the last 15 years on cabergoline treatment in CD, it should be noted that the majority of the published studies are single-center, open-label, non-controlled clinical studies, performed on a limited number of patients (52–56). However, two multicenter studies were also performed; a Swedish prospective study, involving four centers across the country, included only 20 patients followed-up for a short period of time, up to 1.5 months (57), whereas a retrospective French-Belgian study, involving 13 centers across the two countries, was based on a large variability in the definition of disease control, as well as in the hormonal assessments (58). Therefore, a multicenter, international, controlled clinical trial on the use of cabergoline in monotherapy in CD patients is nowadays still lacking, with no on-going trials currently available.

Overall considered, cabergoline represents an effective treatment in patients with CD, particularly in patients with mild disease, in which cabergoline treatment may be considered an interesting approach. Moreover, the oral administration and the good safety profile may be comfortable for CD patients, potentially improving patients' compliance, with a potential positive impact on success rate. Furthermore, cabergoline treatment was occasionally associated with tumor shrinkage, therefore addressing it as an option of interest for patients with visible, especially large, pituitary tumors. Conversely, although an improvement in clinical profile was associated with cabergoline treatment, the variable time needed to reach disease control may represent a potential limitation in cabergoline employment, and therefore it would be reasonable to prefer cabergoline in patients without severe hypercortisolism-related comorbidities, where a prompt hypercortisolism resolution is not required. Finally, due to the occurrence of treatment escape, cabergoline should be preferred for short-term more than for long-term treatment, or in patients available to perform routinely clinical and hormonal assessments, in which a treatment escape may be more easily identified. Notably, the present off-label position of cabergoline in the treatment schedule of the disease may represent a further limitation of the employment of cabergoline in the routine clinical practice of the management of CD.

## Adrenal-Directed Drugs

Adrenal-directed drugs target the adrenal gland, inducing a decrease of cortisol secretion through the inhibition of steroidogenesis, with a specific enzyme inhibitory profile; this is the reason why they are also named “steroidogenesis inhibitors” (4, 60). Considering the actually available drugs, metyrapone is currently under investigation in clinical trials, whereas, considering experimental therapies, three new drugs, osilodrostat, levoketoconazole, and ATR-101, are currently under investigation. Additional adrenal-directed drugs, including ketoconazole, a classical adrenostatic drug currently used in the management of CS, and mitotane, an adrenostatic and adrenolytic drug, currently used in the management of adrenocortical carcinoma (ACC), but occasionally employed in the treatment of severe CS, are not presently under investigation in clinical trials on CS; however, a brief summary of the available evidences on their use in CS patients will be provided.

Table 3 shows the characteristics of current and recent clinical trials, registered in clinicaltrials.gov, on adrenal-directed drugs for the treatment of CD and CS in terms of official study name, identification code, study design, patient number, and start and completion date.

**Table 3:** Characteristics of current and recent clinical trials, registered in clinicaltrials.gov, on adrenal-directed drugs for the treatment of Cushing's disease and Cushing's syndrome in terms of official study name, identification code, study design, patient number, start, and completion date.

**Investigation drug**	**Official study name**	**ClinicalTrials.gov code**	**Study type**	**Estimated patient number**	**Start date**	**Completion date**
Metyrapone	Prospective, Single Arm, Open-label, Multicenter, International Study to assess the effects of metyrapone in patients with endogenous Cushing's Syndrome during a 12-week treatment period followed by an extension period of 24-weeks	NCT02297945	OL; P; MC; Phase III	50*	April 2015	April 2020
Osilodrostat	A Proof of Concept, Open-label, Forced Titration, Multi-center Study to assess the safety/tolerability and efficacy of 10-week treatment of LCI699 Followed by a 12-week treatment period of LCI699 in patients with Cushing's disease	NCT01331239^§^	OL; P; MC; Phase II	31*	March 2011	October 2019
	Phase III, Multi-center, Double-blind, Randomized Withdrawal Study of LCI699 following a 24-week, single-arm, open-label dose titration and treatment period to evaluate the safety and efficacy of LCI699 for the treatment of patients with Cushing's disease	NCT02180217	RW; DB following OL; MC; Phase III	137*	October 2014	December 2019
	A Phase III, Multi-center, Randomized, Double-blind, 48-week Study with an initial 12-week placebo-controlled period to evaluate the safety and efficacy of osilodrostat in patients with Cushing's disease	NCT02697734	R; DB; P; MC; Phase III	73	October 2016	January 2021
	An Open-label, Multi-center, Roll-over Study to assess long term safety in patients with endogenous Cushing's syndrome who have completed a prior Novartis-sponsored osilodrostat (LCI699) study and are judged by the investigator to benefit from continued treatment with osilodrostat	NCT03606408	OL; P; MC; Phase III	180	October 2018	October 2023
Levoketoconazole	An Open Label Study to assess the safety and efficacy of COR-003 (levoketoconazole) in the treatment of endogenous Cushing's syndrome	NCT01838551	OL; P; MC; Phase III	94*	August 2014	November 2018
	A double-blind, placebo-controlled, randomized withdrawal following Open Label Therapy Study to assess the safety and efficacy of levoketoconazole (2S, 4R-ketoconazole) in the treatment of endogenous Cushing's syndrome	NCT03277690	RW; DB following OL; P; MC; Phase III	44	September 2017	August 2020
ATR-101	A Phase 2 Randomized, Double-Blind, Placebo-Controlled Study of ATR-101 for the treatment of Cushing's syndrome	NCT03053271	R; DB; P; MC; Phase II	4*	April 2017	August 2019

*OL, open-label; P, prospective; MC, multicenter; RW, randomized-withdrawal; DB, double-blinded; R, randomized; SC, single-center*.

* *Definitive patient number due to study completion ^§^reported as the identification code for both phase 2 studies*.

### Metyrapone

Metyrapone is an oral steroidogenesis inhibitor, which has been worldwide used as an off-label treatment in the management of CS during the last 40 years; in Europe, it received the official indication for the management of CS by EMA in April 2014, whereas it is still being an off-label treatment in the United States, where FDA officially approved metyrapone only as a diagnostic tool for CS.

Metyrapone is an adrenal enzyme blocker, mainly acting on 11β-hydroxylase, which is responsible for the conversion of 11-deoxycortisol in cortisol and of 11-deoxycorticosterone in corticosterone, an aldosterone precursor, therefore inducing a decrease of glucocorticoid and mineralocorticoid production and secretion (4, 60). Moreover, metyrapone also inhibits the cholesterol side-chain cleavage complex and, to a lesser extent, 17α-hydroxylase and aldosterone synthase (4, 60). Metyrapone has a short half-life (2 h), requiring therefore multiple daily administrations, variably ranging from four to six times per day (4, 60). The inhibition of cortisol synthesis by metyrapone generally induces a compensatory rise in ACTH levels, that may drive glucocorticoid and mineralocorticoid precursors, as well as adrenal androgens, overproduction, which are responsible for typical AEs (4, 60). Metyrapone is formulated as an immediate-release capsule, containing a 250 mg dose, and a maximum daily dose of 6 g/day is suggested.

Metyrapone has been extensively studied for the treatment of CS, showing a revised estimated average remission rate of 75.9% according to a recent meta-analysis (61). In particular, in studies including both exclusively CD and mixed CD and CS populations, metyrapone treatment showed an average remission rate of 71%, ranging from 45.5 to 100% (4). Noteworthy, metyrapone demonstrated a rapid onset of action, being able to decrease UC in the first weeks of treatment in CD and CS patients (4, 60). However, in studies including both exclusively CD and mixed CD and CS populations, a treatment escape was observed in an average of 5.7% of patients, although variably ranging from 0 to 13% among the different studies (4). In CD and CS patients, metyrapone treatment is associated with a general improvement in clinical features, mainly including hypertension, impairment of glucose metabolism, psychiatric disturbances, and muscle weakness (4, 60, 62, 63). In CD patients, no effects on pituitary tumor were reported (62, 63). In studies including both exclusively CD and mixed CD and CS populations, the most frequently reported AEs were hirsutism in women (36.1%), dizziness (30.4%), and arthralgias (13.4%) (4). Noteworthy, AI was reported as an AE only in a British study on 91 CS patients, occurring in 13.3% of patients (64). In a large British study on 195 CS patients, the most frequently reported AEs were gastrointestinal disturbances (23%) and AI (7%) (62), and, in a Spanish study on 62 CS patients, hypertension was reported as an AE, being present in around 48% of patients, although it should be noted that AEs were reported on the overall population, including not only patients treated with metyrapone, but also patients treated with ketoconazole and a combination of metyrapone and ketoconazole, thus potentially biasing the overall AEs rate reported for metyrapone (65). Hypokalemia was reported in two studies, occurring in 6.7–13.6% of patients (64, 66), whereas peripheral oedema was reported in three studies, occurring in 3.2–23.1% of patients (63–65). Although a large amount of evidences is currently available on metyrapone treatment in CS, published studies present limitations. Indeed, the vast majority of these studies are retrospective studies, with only one recent observational, longitudinal, prospective Italian study on 31 CS patients (63), and/or single-center studies, with only the previously mentioned British study on 195 CS patients involving 13 different UK centers (62). Therefore, no prospective, international, multicenter, controlled clinical trials on metyrapone are actually available. Moreover, the currently available reports are heterogeneous for the definition of disease control, with variable treatment response criteria among studies (4, 62, 63).

A prospective, open-label, single-arm, multicenter phase III/IV clinical trial, the PROMPT study (clinicaltrials.gov code: NCT02297945), was started to assess the efficacy and safety of metyrapone treatment in a wide CS population (67). The study was completed on April 2020, and a preliminary, 12-week *interim* analysis has been recently presented in endocrinological meetings (67). Metyrapone was orally administered in a thrice- or four times-daily schedule. In this study, 50 CS patients entered a 12-week, open-label, up-titration period, during which dose up-titration was performed until response to treatment or a maximum dosage of 6 g/day was reached. According to baseline mUC, metyrapone was started at a total daily dose of 750 and 1,500 mg/day in patients with baseline mUC ≤ and >5-fold the ULN, respectively. In patients with mUC normalization (mUC ≤ ULN) or serum cortisol levels between 6 and 12 μg/dl, a stable dose was maintained, whereas in the remaining patients up-titration was resumed. At week 8, patients with mUC >10-fold the ULN were discontinued. At week 12, patients with mUC <2-fold the ULN, but receiving clinical benefit from treatment according to investigators' judgment, entered the 24-week, open-label extension period, whereas in patients with mUC ≥2-fold the ULN metyrapone was discontinued. The primary endpoint of this study was the normalization of mUC after 12 weeks of treatment in patients with CS. Considering the 49 patients included in the efficacy analysis, after 12 weeks of treatment metyrapone at a mean dose of 1,664.9 mg/day induced mUC normalization in 23 (46.9%) patients, whereas a ≥50% mUC decrease was reached in additional 16 (32.7%) patients, with a total success rate of 79.6%. Overall, a median decrease of 73.5% was observed in median mUC after 12 weeks of treatment. Noteworthy, mean time to mUC normalization was 34 days. A decrease in LNSC was also observed, with LNSC normalization in 11 (22.4%) patients, whereas an increase in testosterone levels was observed in female patients. Regarding clinical profile, a decrease in blood pressure, total cholesterol, and plasma insulin levels was observed, as well as an improvement in quality of life. Noteworthy, considering the 32 patients taking antihypertensive medications, 10 (31.2%) patients were able to reduce medications, whereas five (15.6%) patients required an increase. The most frequently reported AEs, grouped by category and considering the overall population, were gastrointestinal disorders (30%), general disorders and administration site conditions (26%), and metabolism and nutrition disorders (20%); no details were provided regarding the AEs included in each category, except for the presence of nausea in the gastrointestinal disorders category, occurring in 24% of patients. AI was reported only in six (12%) patients. Treatment discontinuation occurred in three (6%) patients, because of AEs in one (2%) patient. No patients discontinued treatment because of lack of efficacy (67). Definitive data are not yet available.

Overall considered, metyrapone represents an effective treatment for CD and CS, and its rapid action may lead to consider it as an interesting approach in patients requiring prompt relief from hypercortisolism-related comorbidities. However, metyrapone does not affect the pituitary tumor, which should be properly monitored in case of long-term treatment. Moreover, although the oral administration may be comfortable for patients, the multiple daily administrations may discourage patients from properly following treatment schedule, with a potential negative impact on success rate. Furthermore, as in women hirsutism was reported, metyrapone should be preferred in men. Moreover, due to the potential occurrence or worsening of hypokalemia, metyrapone should be preferred in patients without severe or with well-controlled hypokalemia, and careful monitoring of potassium levels should be performed during the clinical follow-up. Finally, due to the possible occurrence of treatment escape, metyrapone should be preferred for short-term more than for long-term treatment, or in patients available to perform routinely clinical and hormonal assessments, in which a treatment escape may be more easily identified.

### Ketoconazole

Ketoconazole is an oral adrenal steroidogenesis inhibitor, which has been worldwide used as an off-label treatment in the management of CS during the last 30 years; in Europe, it received the official license for the treatment of CS in adults and in adolescents above the age of 12 years by EMA in November 2014, whereas it is still an off-label treatment in the United States, where no official approval for ketoconazole in CS treatment has been granted.

Ketoconazole, which is currently available as a 50/50 racemic mixture of two enantiomers, levoketoconazole (2S,4R stereoisomer) and dextroketoconazole (2R,4S stereoisomer), is an adrenal enzyme blocker, acting on several steroidogenesis enzymes, including the cholesterol side-chain cleavage complex, 17,20-lyase, 11β-hydroxylase, and 17β-hydroxylase, therefore inducing a decrease in glucocorticoid, mineralocorticoid, and adrenal androgen production and secretion (4, 60). Noteworthy, ketoconazole was also found to inhibit ACTH release in both *in vitro* studies on AtT20 mouse corticotroph tumor cell line and on human corticotroph tumor cell cultures and *in vivo* in rats, thus potentially suggesting a double pharmacodynamic action of potential use in CD patients (4). Ketoconazole presents a relatively short half-life (3.3 h), requiring therefore a twice- or thrice-daily administration schedule (4, 60). Noteworthy, ketoconazole impairs not only adrenal, but also gonadal steroidogenesis, in particular with a negative effect on testicular androgen production, thus potentially leading to male hypogonadism (4, 60). Moreover, ketoconazole inhibits liver enzymes involved in metabolism of chemical substances not normally found or expected to be present in human organism, therefore favoring the occurrence of liver damage (4, 68). Ketoconazole is formulated as an immediate-release tablet, containing a 200 mg dose, and a maximum total daily dose of 1,200 mg/day is suggested.

Ketoconazole was originally conceived as an antifungal drug, being able to interfere with the fungal sterol synthesis of ergosterol, a key element of fungal cell membranes, through the enzymatic blockade of several fungal steroidogenesis enzymes (69). However, later *in vitro* and *in vivo* studies showed that ketoconazole was also able to inhibit adrenal steroid production (69), therefore leading to its clinical use in the treatment of CS. Indeed, ketoconazole has been widely studied in the treatment of CS, showing a revised estimated average remission rate of 71.1% according to a recent meta-analysis (61). In particular, in studies including both exclusively CD and mixed CD and CS populations, ketoconazole treatment showed an average remission rate of 64.3%, ranging from 45 to 93% (4). Ketoconazole demonstrated a rapid onset of action, leading to a decrease in UC in the first weeks of treatment, both in CD and CS patients (4, 60). However, in studies including both exclusively CD and mixed CD and CS populations, a treatment escape was observed in an average of 10.9% of patients, although variably ranging from 7.1 to 13.2% among the different studies (4). In both CD and CS patients, ketoconazole treatment is associated with a general improvement in clinical features, mainly overweight or obesity, hypertension, impairment of glucose metabolism, muscle weakness, and hypokalemia (4, 60). In CD patients, no effects on pituitary tumor were reported (4). In studies including both exclusively CD and mixed CD and CS populations, the most frequently reported AEs were hepatotoxicity, represented by liver enzymes elevation (14.5%), gastrointestinal disturbances (12.9%), and AI (11.9%) (4). Noteworthy, male hypogonadism was reported in healthy subjects after ketoconazole administration (70), but, in CS patients, only a worsening of gynecomastia, a potential sign of hypogonadism, was reported in a single study as an AE in about 17% of men with CS (71). Furthermore, although ketoconazole is generally considered as a drug potentially affecting cardiac QT, actually QT prolongation was never reported as an AE (4), and an Italian study focusing on potential disorders of cardiac rhythm during ketoconazole treatment in CD patients did not identify any QT alterations after ketoconazole administration (72).

Although a large amount of evidences is currently available on ketoconazole treatment in CS, published studies present limitations. Indeed, all the available studies are retrospective and single-center studies, with the relevant exception of a large retrospective French study on 200 CS patients involving 14 different centers (73); however, due to the retrospective nature of the study, the efficacy and safety report may have been influenced by the heterogeneity in clinical evaluations performed in each center. No prospective, international, multicenter, controlled clinical trials on ketoconazole are actually available, neither they are currently on-going.

Overall considered, ketoconazole represents an effective treatment for CD and CS, and its rapid action may lead to consider it as an interesting approach in patients requiring prompt relief from hypercortisolism-related comorbidities. However, although debated, ketoconazole seems not to affect the pituitary tumor, which should be properly monitored in case of long-term treatment. Moreover, although the usual twice-daily, oral administration may be comfortable for patients, a more frequent administration may be required at higher doses, therefore potentially discouraging patients from properly following treatment schedule, with a potential negative impact on success rate. Furthermore, as male hypogonadism has been occasionally reported, ketoconazole should be preferred in women and in men with clearly normal androgen production, with a close monitoring of gonadal function. Moreover, as hepatotoxicity was reported, ketoconazole should be preferred in patients without severe liver disease, and a strict monitoring should be performed in patients already experiencing liver damage. Finally, due to the occurrence of treatment escape, ketoconazole should be preferred for short-term more than for long-term treatment, or in patients available to perform routinely clinical and hormonal assessments, in which a treatment escape may be more easily identified.

### Osilodrostat

Osilodrostat is a novel oral steroidogenesis inhibitor, currently under clinical evaluation for CS treatment and officially licensed by EMA on January 2020 for the treatment of endogenous CS in adults and by FDA on March 2020 for the treatment of CD patients who either cannot undergo pituitary surgery or have undergone pituitary surgery but still have the disease.

Osilodrostat potently inhibits the adrenal enzymes aldosterone synthase, which is responsible for the conversion of corticosterone to aldosterone, and 11β-hydroxylase, which is responsible for the conversion of 11-deoxycortisol to cortisol and of 11-deoxycorticosterone to corticosterone, therefore inducing a decrease in glucocorticoid and mineralocorticoid production and secretion (74). In particular, an experimental direct comparison of osilodrostat with metyrapone and ketoconazole, two classical adrenal steroidogenesis inhibitors, has shown that osilodrostat inhibits *in vitro* cortisol production more potently than both metyrapone (IC_50_ 0.0347 μM vs. 0.0678 μM) and ketoconazole (IC_50_ 0.0347 μM vs. 0.621 μM) in human adrenocortical HAC15 cell cultures, suggesting that lower doses of osilodrostat compared to metyrapone and ketoconazole may be sufficient to reach the same efficacy, despite the variable results observed in human adrenal cell cultures deriving from cortisol-producing adrenal hyperplasias, adrenal adenomas, and ACC (75). Moreover, osilodrostat has shown a longer half-life compared with both metyrapone (4 vs. 2 h) and ketoconazole (4 vs. 3.3 h), allowing therefore a twice-daily administration (4, 76, 77). Osilodrostat is formulated as three different immediate-release tablets, containing 1, 5, and 10 mg doses, respectively, with suggestion of maximum total dose of 60 mg/day.

Due to its prevalent action on the mineralocorticoid pathway, osilodrostat was originally conceived as an antihypertensive drug, aiming to offer an additional approach to classical antialdosteronic agents, including spironolactone and eplerenone, as the latter presents a poor tolerance profile, and the former has a lower potency compared to spironolactone (77). In a first phase I, double-blind, placebo-controlled study on healthy normotensive subjects, osilodrostat administration for 7 days at doses of 1–10 mg/day was associated with a decrease in urinary and plasma aldosterone of 22–83% and 27–44%, respectively (76). Interestingly, an inhibition of cortisol response to ACTH stimulation was reported at a dose of 3 mg/day, with loss of aldosterone synthase inhibition selectivity already at doses of 1 mg/day (77). Moreover, some healthy subjects experienced weight loss (25%), postural tachycardia (25%), and mild hyponatremia (33.3%), all of which considered to be related to the inhibition of aldosterone secretion (76, 77). A further phase I study on healthy subjects confirmed that osilodrostat treatment at doses >3 mg/day was associated with a decrease of baseline and ACTH-stimulated cortisol secretion, and an increase of baseline and ACTH-stimulated 11-deoxycortisol levels, together with an increase in plasma ACTH levels (78). On the basis of these preliminary evidences, osilodrostat treatment was further investigated in hypertensive patients, confirming a decrease of ACTH-stimulated cortisol levels in patients treated with osilodrostat, in a dose-dependent and time-dependent manner (79).

A first phase II, proof-of-concept, single-arm, multicenter clinical trial, the LINC1 study (clinicaltrials.gov code: NCT01331239), was started to assess the efficacy and safety of osilodrostat treatment in CD patients for a period of 10 weeks (80). Osilodrostat was orally administered in a twice-daily schedule. In this study, 12 patients with recurrent CD after pituitary surgery and mUC >1.5-fold the ULN entered a 10-week, open-label, up-titration period, during which, starting with an initial dose of 4 mg/day, dose up-titration was performed every 2 weeks until mUC normalization (mUC ≤ ULN) or the achievement of maximum dose of 100 mg/day, followed by a 2-week, treatment-withdrawal period. During the up-titration period, if mUC normalized, dose was maintained until week 10, whereas if mUC re-increased above the ULN after previous normalization, dose up-titration was resumed. The primary endpoint of this study was the proportion of patients with mUC ≤ ULN or who had a ≥50% mUC decrease from baseline at week 10. After 10 weeks of treatment, osilodrostat induced mUC normalization in 11 (91.7%) patients and a ≥50% mUC decrease, without normalization, in the remaining (8.3%) patient. In nine (75%) patients, mUC normalization was obtained with doses ≤ 20 mg/day, whereas the remaining three (25%) patients reached mUC normalization with 30, 40, and 100 mg/day, respectively. A notable mUC decrease was obtained after 4 weeks, with a significant decrease of 86% in mUC after 10 weeks of treatment; after treatment discontinuation, mUC returned above the ULN in the entire cohort of patients. Moreover, serum cortisol and plasma aldosterone levels significantly decreased, whereas plasma ACTH, 11-deoxycortisol, and 11-deoxycorticosterone levels significantly increased during treatment. In the eight women, serum testosterone levels significantly increased, with six (75%) women with normal baseline serum testosterone levels and one (12.5%) with increased baseline serum testosterone levels experiencing levels above the ULN after 9 weeks of treatment. The remaining patient had fluctuating testosterone levels during the study. The four men experienced an increase, although non-significant, in mean testosterone levels, reaching the lower limit of normal range (80). Regarding clinical profile, after 10 weeks of treatment, a decrease in blood pressure was observed, and two (66.7%) of three initially hypertensive patients became normotensive, as well as a small, but notable decrease in glycated hemoglobin (HbA1c) was noted, both in total population and in patients with HbA1c levels ≥6.5%. However, patients experienced a slight increase in body weight (80). Regarding the safety profile, the most frequently reported AEs were fatigue (58.3%), nausea (41.7%), diarrhea (25%), headache (25%), hypokalemia (25%), muscle spasms (25%), and vomiting (25%). In four (33.3%) patients, AEs probably related to AI or cortisol withdrawal syndrome occurred, requiring dose down-titration or transient treatment interruption; in one case (25%), glucocorticoid replacement was required. Hypokalemia was also identified in four (33.3%) patients, although it was reported as an AE only in three (25%) patients, which received potassium supplementation normalizing potassium levels. One (8.3%) patient experienced oedema, together with a relevant weight gain, associated with a dramatic increase of plasma levels of 11-deoxycorticosterone. Only one serious AE was reported, with patient experiencing severe anemia, palpitations, and chest pain, secondary to the reactivation of a Takayasu arteritis, probably consequence of cortisol withdrawal; the AE resolved with blood transfusion and was not considered related to osilodrostat treatment (80). Noteworthy, no treatment discontinuations occurred.

A second phase II, proof-of-concept, open-label, LINC1 study-expansion by protocol amendment, multicenter clinical trial, the LINC2 study (clinicaltrials.gov code: NCT01331239), was performed for a period of 22 weeks, with an optional extension phase of 48 weeks, to assess the efficacy and safety of osilodrostat treatment in CD patients for a longer treatment period (81). Osilodrostat was orally administered in a twice-daily schedule. Overall, 19 CD patients with mUC > ULN, comprising two cohorts, were enrolled: the “follow-up cohort”, including four of the 12 patients of the LINC1 study, and the “expansion cohort”, including 15 osilodrostat-naïve patients. In the follow-up cohort, osilodrostat was started at the penultimate effective and tolerable dose assumed in the LINC1 study, whereas in the expansion cohort osilodrostat was started at a dose of 4 mg/day. The patients enrolled in both cohorts entered a 10-week, open-label, up-titration period, during which a dose up-titration was performed every 2 weeks until mUC normalization (mUC ≤ ULN) or a maximum dose of 60 mg/day was reached, followed by a 12-week, open-label, maintenance period. During the up-titration period, if mUC normalized, the dose was maintained until week 22, whereas if mUC re-increased above the ULN after previous normalization, dose up-titration was resumed. Differently from LINC1 study (80), in LINC2 study the maximum acceptable daily dose was 60 mg/day (81). At the end of the 22-week period, patients with mUC normalization or receiving clinical benefits from the treatment were offered to continue for a further 48-week extension period. The primary endpoint of this study was the proportion of responder patients, including patients with full control, defined as patients with mUC ≤ ULN, and patients with partial control, defined as patients without mUC ≤ ULN, but with a ≥50% mUC decrease from baseline levels, and the proportion of non-responder uncontrolled patients, defined as patients without mUC ≤ ULN and without a ≥50% mUC decrease from baseline levels, at 10 and 22 weeks. During the up-titration period, two (10.5%) patients discontinued treatment, whereas the remaining 17 (89.5%) completed the 22-week period. Considering the overall population, after 10 weeks of treatment, osilodrostat induced mUC normalization in 16 (84.2%) patients and a ≥50% mUC decrease in one (5.3%) additional patient, with a total success rate of 89.5%. After 22 weeks of treatment, osilodrostat induced mUC normalization in 15 (78.9%) patients, whereas a decrease of 48.6 and 47.4% in mUC, respectively, was observed in the two (21.1%) remaining patients, therefore with a total success rate of 78.9%. A significant drop in mUC was observed after 4 weeks of treatment, remaining stably reduced till week 22. Moreover, in patients reaching week 22, a decrease in serum cortisol, morning salivary cortisol, LNSC, and plasma aldosterone levels and an increase in plasma ACTH, 11-deoxycortisol, and 11-deoxycorticosterone levels were observed. In the 14 women enrolled in the study, testosterone levels significantly increased, with five (35.7%) women with increased baseline testosterone levels presenting testosterone levels above the normal range, during the study. In the 12 women reaching week 22, nine (75%) had testosterone levels above the normal range at the end of the study. Moreover, in men, testosterone levels, whose mean baseline values were below the normal range, increased till, reaching the normal range (81). Regarding clinical profile, after 22 weeks of treatment, a decrease in fasting serum glucose, HbA1c, cholesterol, and triglyceride levels was observed, whereas a minor decrease was reported in body weight and systolic blood pressure. Only six (31.6%) patients had a measurable pituitary tumor at pituitary MRI at baseline and at the last follow-up, and among these patients, two (33.3%) presented an increase of tumor maximum diameter, not considered as clinically meaningful, after 22 weeks (81). Regarding safety profile, the most frequently reported AEs were nausea (31.6%), diarrhea (31.6%), asthenia (31.6%), and AI (31.6%) for the overall population, whereas, considering women, hirsutism and/or acne were also frequently reported (28.6%), and were associated with increased testosterone levels. A mild hypokalemia was registered in nine (47.4%) patients, being reported as an AE in one (5.3%) patient and being treated with potassium supplementation in two (10.5%) patients. Three serious AEs were reported, two in one patient (acute gastroenteritis with dehydration and QT prolongation) and one in a different patient (uncontrolled CD). Treatment discontinuation occurred in two (10.5%) patients, because of AEs in one (5.3%) and personal decision not to continue treatment in the extension period at the end of week 22 in the other one (5.3%) patient. Noteworthy, no treatment discontinuation due to lack of efficacy occurred (81).

A phase III, double-blind, randomized-withdrawal following an open-label, single-arm period, multicenter clinical trial the LINC3 study (clinicaltrials.gov code: NCT02180217), was started to assess the efficacy and safety of osilodrostat treatment on a large population of CD patients for a period of 48 weeks (82). Osilodrostat was orally administered in a twice-daily schedule. In this study, 137 patients with mUC ≥1.5-fold the ULN entered a 12-week, open-label period, during which, starting at an initial dose of 4 mg/day, a dose up-titration was performed every 2 weeks until mUC normalization (mUC ≤ ULN) or a maximum dose of 60 mg/day was reached, followed by a 12-week, maintenance period. During up-titration and maintenance periods, if mUC normalized, the dose was maintained stable, whereas if mUC re-increased above the ULN after previous normalization, dose up-titration was resumed. At week 24, patients with mUC normalization without dose up-titration after week 12 were considered eligible for an 8-week, double-blind, randomized-withdrawal period, whereas non-eligible patients continued osilodrostat in an 8-week, open-label period. Of the 137 enrolled patients, 71 (51.8%) patients were considered eligible for randomization and double-blindly randomized to osilodrostat (36 patients) or placebo (35 patients), whereas of the remaining 66 (48.2%) patients, 19 (13.9%) discontinued treatment and 47 (34.3%) continued open-label osilodrostat treatment. At week 34, both randomized and non-randomized patients started a further 14-week, open-label period of osilodrostat treatment. The primary endpoint of this study was the proportion of participants who had been randomly assigned to treatment in the randomized-withdrawal phase who maintained mUC normalization during osilodrostat therapy or matching placebo at the end of the 8-week, randomized-withdrawal period without any dose increase during this period. At the end of the randomized-withdrawal period, 31 (86.1%) of 36 patients treated with osilodrostat and 10 (29.4%) of 34 patients treated with placebo maintained mUC normalization without dose up-titration during the first 24 weeks. Moreover, at week 12, osilodrostat induced mUC normalization in 98 (71.5%) patients regardless of dose up-titration, whereas at week 24, osilodrostat induced mUC normalization in 72 (52.6%) patients without dose up-titration and in 93 (67.9%) patients regardless of dose up-titration. Finally, at week 48, osilodrostat induced mUC normalization in 91 (66.4%) patients regardless of dose up-titration. Moreover, excluding patients randomized to placebo, 64 (66%) patients maintained mUC normalization for at least 6 months after the first mUC normalization. Considering the overall population, a significant decrease in mUC was observed in the first 12 weeks, as well as in mean serum cortisol and LNSC levels, all of which remained stably below the baseline values throughout the study. Noteworthy, mean serum cortisol levels not only decreased compared with baseline, but they also reached the normal range and remained in the normal range throughout the study. Furthermore, a decrease in plasma aldosterone levels and an increase in plasma ACTH, 11-deoxycortisol, and 11-deoxycorticosterone levels were observed. Notably, in patients randomized to placebo, the increase in plasma ACTH and 11-deoxycortisol levels reversed during the randomization period. An increase in testosterone levels was observed in the whole cohort in the first 12 weeks, whereas in the subsequent study periods no further increases were observed. In women, mean testosterone levels increased from the normal range to the ULN, whereas in men, testosterone levels increased from the lower limit of normal range at baseline to the mid-normal range during treatment, with men with hypogonadism at baseline reaching normal testosterone levels throughout the study (82). Regarding clinical profile, at week 48, a decrease in body weight, blood pressure, total and LDL cholesterol, fasting serum glucose, and HbA1c, with a minor decrease in high-density lipoprotein (HDL) cholesterol, together with improvement of quality of life and depression status, was observed; the clinical improvement mainly occurred in the first 12 weeks. Considering patients with measurable tumor at pituitary MRI both at baseline and at 24 weeks, 20 (30.3%) and 19 (28.9%) of 66 patients had a ≥20% increase and decrease, respectively, in tumor volume at 24 weeks; similar evidences were reported in patients with measurable tumor at pituitary MRI both at baseline and at 48 weeks, when 24 (37.5%) and 21 (32.8%) of 64 patients had a ≥20% increase and decrease, respectively, in tumor volume (82). Regarding safety profile, the most frequently reported AEs were nausea (41.6%), headache (33.6%), and fatigue (28.5%), together with hirsutism (11.3%), acne (11.3%), and hyperthrychosis (0.9%) in women. AEs were also grouped in categories of special interest, including hypocortisolism-related AEs (51.1%), adrenal hormone precursors increase-related AEs (42.3%), mainly represented by hypokalemia (13.1%) and hypertension (12%), QT prolongation (3.6%), pituitary tumor enlargement (2.9%), and arrhytmogenic-potentially-related episodes (0.7%). Treatment discontinuation occurred in 24 (17.5%) patients, because of AEs in 15 (10.9%) patients. Noteworthy, no treatment discontinuation because of lack of efficacy occurred (82).

Following LINC studies, an open-label, multicenter, roll-over study (clinicaltrials.gov code: NCT03606408) was started at the end of 2018, in order to allow patients to continue their treatment even after official study completion, up to a maximum of 180 patients previously treated with osilodrostat.

Noteworthy, in October 2016, concomitantly to the LINC3 study, a second phase III, randomized, double-blind with an initial placebo-controlled period, multicenter study, the LINC4 study (clinicaltrials.gov code: NCT02697734), was started to evaluate the efficacy and safety of osilodrostat treatment in CD patients with a different study design for a period of 48 weeks. Osilodrostat is orally administered in a twice-daily schedule. This study expects to enroll 73 CD patients, which enter a 12-week, double-blind, randomized period, during which they are randomly assigned to placebo or treatment group, followed by an open-label period. The primary endpoint of this study is the superiority of osilodrostat compared to placebo in achieving a complete response (mUC ≤ ULN) after 12 weeks of treatment. Expected study completion date is January 2021, but no preliminary results are currently available.

Overall considered, osilodrostat seems to be an effective treatment for CD and CS, and its potent and rapid action may lead to consider it as an interesting approach for the treatment of patients with CD and CS, including those with severe disease and/or requiring prompt relief from hypercortisolism-related comorbidities. Moreover, the twice-daily, oral administration and the good safety profile may be comfortable for patients, potentially improving treatment compliance, with a potential positive impact on success rate. However, osilodrostat effects on the pituitary tumor are yet to be fully elucidated, so it should be properly monitored in case of long-term treatment. Moreover, due to the potential occurrence or worsening of hypokalemia, osilodrostat should be preferred in patients without severe or with well-controlled hypokalemia, and careful monitoring of potassium levels should be performed during the clinical follow-up. Furthermore, due to the occurrence of cortisol withdrawal syndrome, or AI, probably due to the great potency of osilodrostat, the treatment should be associated with an early and frequent clinical monitoring especially in patients with mild disease and particularly in the first weeks of treatment.

### Levoketoconazole

Levoketoconazole is a novel, oral steroidogenesis inhibitor, currently under clinical evaluation for CS treatment.

Levoketoconazole is the cis-2S,4R stereoisomer of the classical racemic ketoconazole, showing therefore a similar enzymatic inhibitory profile of ketoconazole, with a prevalent action on cholesterol side-chain cleavage complex, 11β-hydroxylase, 17α-hydroxylase, and aldosterone synthase, therefore inducing a decrease in glucocorticoid, mineralocorticoid, and adrenal androgen production and secretion (68, 83). In experimental models, compared with dextroketoconazole, levoketoconazole displayed a higher affinity for 11β-hydroxylase and 17α-hydroxylase enzymes, with consequent inhibition at lower doses of enzyme activity (68). Moreover, levoketoconazole inhibited liver enzymes at concentrations about 12-fold higher than those of dextroketoconazole (68). In human AD-293 cell line, derived from embryonic kidney HEK-293 cell line, and expressing steroidogenesis enzymes, levoketoconazole inhibited the enzymatic activities of 11β-hydroxylase, 17α-hydroxylase, and 21-hydroxylase more potently compared to racemic ketoconazole (84). Furthermore, levoketoconazole showed a half-life of around 2.6–4.8 h, thus requiring a twice-daily administration (85). Levoketoconazole is formulated as an immediate-release 150 mg tablet and it is still only available in experimental studies.

Due to the suboptimal safety profile of ketoconazole and to its racemic composition, one of the first experimental approaches to improve its feasibility in human employment was to evaluate the specific profile of enzymatic affinity for each of the two ketoconazole stereoisomers (68). As previously reported in experimental models, levoketoconazole showed a higher potency toward adrenal steroidogenesis enzymes inhibition and a lower potency toward liver enzymes inhibition compared to dextroketoconazole (68), thus suggesting it as a potential candidate for clinical investigation. Surprisingly, levoketoconazole was thereafter tested as a potential new antidiabetic drug (86). Indeed, higher cortisol levels are reported in diabetic patients compared with healthy subjects (87), therefore suggesting a hypothetical improvement in glycemic control due to the decrease in circulating cortisol levels (86). However, clinical studies on levoketoconazole in diabetes treatment were unsuccessful, as conflicting results on glycemic control were reported, together with early study terminations due to safety concerns (86). Therefore, new preclinical studies were started, showing in animal models that levoketoconazole reduced by 50% serum corticosterone levels at a dose of 100 mg/kg, significantly lower than those required by racemic ketoconazole, with a maximum corticosterone suppression within 4 h, maintained over 24 h with a 200 mg/kg dose (84). Due to the positive findings in animals, phase I studies on healthy subjects were performed, showing a good safety profile, as patients mainly experienced headache, nausea, and dizziness as AEs, and the absence of liver enzymes increase during treatment (84, 85), supporting the hypothesis of a reduced hepatotoxicity compared to racemic ketoconazole.

A first phase III, open-label, multicenter clinical trial, the SONICS study (clinicaltrials.gov code: NCT01838551), was started to assess the efficacy and safety of levoketoconazole treatment in CS patients for a period ranging from 12.5 to 16.8 months according to the initial up-titration period duration (88). Levoketoconazole was orally administered in a twice-daily schedule. In this study, 94 patients with mUC ≥1.5-fold the ULN, including 80 CD patients, entered an up-titration period of 2–21 weeks, during which, starting at an initial dose of 300 mg/day, a dose up-titration was performed every 2 weeks until mUC normalization (mUC ≤ ULN) or a maximum dose of 1,200 mg/day was reached, followed by a 6-month, open-label, stable-dose, maintenance period, during which they were treated with stable doses, unless a dose change was needed to maintain disease control or in response to safety and tolerability issues. During up-titration period, if mUC normalized, patients were maintained at stable doses, whereas if mUC re-increased above the ULN after previous normalization, dose up-titration was resumed. At the end of maintenance period, patients were offered to enter a further 6-month, open-label, extension period. The primary endpoint of this study was the proportion of patients reaching mUC normalization at the end of the 6 months of the maintenance period without a dose up-titration during maintenance period. During up-titration period, 17 (18.1%) patients discontinued treatment, and the remaining 77 (81.9%) patients entered the maintenance period. After the 6 months of the maintenance period, levoketoconazole induced mUC normalization in 29 (30.8%) patients without dose up-titration and in 34 (36.1%) patients regardless of dose up-titration. Considering as responders not only patients with mUC normalization, but also patients without mUC normalization, but with a >50% mUC decrease compared with baseline, 43 (45.7%) patients showed a response to treatment. Considering the 55 patients who completed the maintenance period and performed mUC assessment both at baseline and at the end of maintenance period, mUC normalization rates were higher, both without dose up-titration (52.7%) and regardless of dose up-titration (61.8%). Moreover, considering the same study population, 43 patients reached mUC normalization or a >50% mUC decrease compared with baseline, therefore reaching a maximum success rate of 78.2%. Mean mUC decreased significantly in the up-titration period, remaining lower than baseline throughout the study. LNSC levels significantly decreased from baseline to the end of maintenance period, although normalization occurred only in four (4.2%) patients, whereas, in CD patients, plasma ACTH levels increased during up-titration period and remained elevated throughout the study. Moreover, mean testosterone levels significantly decreased in women, whereas in men a slight, although non-significant, increase was observed (88). Regarding clinical profile, after the 6 months of the maintenance period, a significant decrease in body weight, total, LDL, and HDL cholesterol, fasting serum glucose, and HbA1c, as well as an improvement in quality of life and depressive status, in peripheral oedema, and in acne and hirsutism in women, were observed (88). Levoketoconazole effects on pituitary tumor in CD patients were not evaluated in this study. Regarding safety profile, the most frequently reported AEs were nausea (32%), headache (28%), and peripheral oedema (19%). Hypertension was reported in 16 (17%) patients, although no increase in mean systolic and diastolic blood pressure was observed (88). AEs were also grouped in categories of special interest, including liver related-AEs (7.4%), QT prolongation (5.3%), and AI (3.2%). However, an increase in liver enzymes, including alanine aminotransferase (ALT), γ-glutamyltransferase (GGT), and aspartate aminotransferase (AST), was reported as an AE in 14.9, 12.8, and 11.7% of patients, respectively. Moreover, an increase in ALT values within 1- to 3-fold the ULN, regardless whether it was reported as an AE or not, was also registered in 39 (41.5%) patients, and mainly occurred within 8 weeks from the start of maintenance doses. In diabetic patients, some AEs, including nausea, vomiting, and urinary trait infections, were more frequent, probably due to the specific burden of diabetes or due to antidiabetic drugs (89). Treatment discontinuation occurred in 33 (35.1%) patients, because of AEs in 12 (12.8%) and lack of efficacy in seven (7.4%) patients (88).

A phase III, double-blind, placebo-controlled, randomized-withdrawal following an open-label period, multicenter clinical trial, the LOGICS study (clinicaltrials.gov code: NCT03277690), was started in September 2017 to assess the efficacy and safety of levoketoconazole treatment in CS patients for a period ranging from 16 to 38 weeks, according to the cohort of patients. The study expected to enroll 44 CS patients, both levoketoconazole-naïve and previously treated with levoketoconazole during the SONICS study, the SONICS-completers. Levoketoconazole-naïve and SONICS-completers not treated with levoketoconazole during the 12 weeks before enrollment or treated without the achievement of mUC normalization (mUC ≤ ULN), entered a 14 to 19-week, open-label, up-titration/maintenance period, during which a dose up-titration was performed until mUC normalization or achievement of a maximum dose of 1,200 mg/day. If mUC normalized, the dose was maintained until the end of up-titration/maintenance period, whereas if mUC re-increased above the ULN after previous normalization, dose up-titration was resumed. Patients completing the up-titration period with sustained mUC normalization for at least 4 weeks and SONICS-completers treated with levoketoconazole during the 12 weeks before enrollment with the achievement of mUC normalization entered a double-blind, stable-dose, randomization period, when they were randomized to levoketoconazole or placebo at stable doses for a period of ~8 weeks, with a maximum of 9.5 weeks. In case of significant worsening of clinical conditions during randomization period, randomized, double-blind treatment assignment was discontinued, and patients directly continued in an 8 to 9.5-week, open-label, restoration period, in which levoketoconazole treatment was resumed. In case of stable mUC and clinical conditions during randomization period, patients continued to an 8 to 9.5-week, double-blind, restoration period, in which patients double-blindly continued levoketoconazole or placebo, according to their previous assignment, and double-blindly added the previously non-assigned treatment in a fast up-titration schedule. The primary endpoint of this study was the number of subjects with loss of therapeutic response to levoketoconazole upon withdrawing to placebo compared with the proportion of subjects with loss of therapeutic response upon continuing treatment with levoketoconazole. The loss of therapeutic response was considered as the mUC rebound after normalization during randomization period, defined as mUC ≥1.5-fold the ULN or an increase >40% in mUC compared to the start of randomization period, or as the occurrence of rescue criteria, requiring open-label treatment, during randomization period. The study was completed on August 2020, but no preliminary data are available.

Clinical studies reporting a direct comparison between the classical racemic ketoconazole and levoketoconazole in terms of both therapeutic efficacy and safety profile are currently lacking, mainly because levoketoconazole is still under clinical investigation and therefore not available in the real-world clinical setting. Moreover, although referring to an international, multicenter, prospective clinical trial, the reported evidences on levoketoconazole treatment for CS rely on a single published study (88), as no preliminary data of the LOGICS study are currently available. Conversely, although extensively evaluated in CS treatment in retrospective studies, racemic ketoconazole was never investigated in prospective, multicenter clinical trials, therefore not allowing definitive conclusions on its employment in clinical settings (4, 60). Indeed, the differences in study design among clinical studies on levoketoconazole and racemic ketoconazole are crucial in the comparative evaluation of their treatment efficacy and safety. The phase III clinical trial performed on levoketoconazole is characterized by strict criteria in terms of definition and evaluation of treatment efficacy, up-titration and down-titration schedule, AEs reporting and management, and choice of treatment discontinuation (88), whereas the retrospective studies, and in particular the multicenter retrospective studies, generally performed on racemic ketoconazole, are not characterized by strict criteria, which are usually also non-homogeneous across involved clinical centers, as clinical practice is not standardized for each center, and over time, as the availability of new evidences and findings may change clinical practice. Therefore, it cannot be excluded, as previously reported for pasireotide, that evidences from the phase III study on levoketoconazole may potentially underestimate its efficacy and safety in the real-world experience, whereas the evidences from the retrospective studies on racemic ketoconazole may potentially overestimate its therapeutic efficacy and safety. In conclusion, further evidences on levoketoconazole, as well as prospective, controlled evidences on racemic ketoconazole, are required to perform a comparison between the two study drugs, even in the absence of comparative studies.

Overall considered, levoketoconazole seems to be an effective treatment in CD and CS, and its rapid action may lead to consider it as an interesting approach in patients requiring prompt relief from hypercortisolism-related comorbidities. Moreover, the twice-daily oral administration may be comfortable for patients, potentially improving patients' compliance, with a potential positive impact on success rate. Furthermore, as hypogonadism was not observed in men, levoketoconazole may be considered a potentially feasible treatment in both sexes. However, levoketoconazole seems not to affect the pituitary tumor, which should be properly monitored in case of long-term treatment. As hepatotoxicity was reported, levoketoconazole should be preferred in patients without severe liver disease, and a strict monitoring should be performed in patients already experiencing liver damage. Likewise, due to the occasional occurrence of hypertension, levoketoconazole should be preferred in patients without severe hypertension, and a strict monitoring should be preferred in patients already experiencing hypertension, as well as optimizing antihypertensive therapy before starting of treatment.

### ATR-101 (Nevanimibe)

ATR-101 is a novel, oral adrenal-directed drug, currently under clinical evaluation for the treatment of CS, ACC, and congenital adrenal hyperplasia.

Unlike currently available adrenal-directed drugs, which exert their effects on steroidogenesis enzymes, ATR-101 exerts its action on acyl coenzyme A: cholesterol acyltransferase 1 (ACAT1), a transmembrane enzyme involved in cholesterol metabolism (90, 91). ACAT1 catalyzes the synthesis of cholesterol esters from free cholesterol and fatty acids, protecting cells from the toxicity of excessive intracellular free cholesterol levels and inducing the storage of cholesterol esters products into specific cytosolic droplets (90). ACAT1 is expressed ubiquitously with highest expression observed in macrophages, where it is involved in the “foam cells” formation during atherosclerosis, and in adrenal glands, where it creates a substrates reservoir for steroid biosynthesis (91, 92). ATR-101 is formulated as immediate-release tablets, containing 125, 250, and 500 mg doses, and it is still only available in experimental studies.

ACAT1 was originally considered as a potential target for the development of new antiatherosclerotic agents, acting as ACAT1 inhibitors and therefore hypothetically preventing lipid accumulation in macrophages and the formation of “foam cells” (93). However, preliminary studies in animals showed unsatisfactory results, mainly because effective doses were associated with adrenolytic effects (93). In particular, in a study on 18 beagle dogs, ATR-101 induced adrenocortical degeneration and/or necrosis in adrenal zona fasciculata and reticularis, with consequent AI (94). Interestingly, adrenocortical degeneration occurred at any dose level, whereas the appearance of AI signs and symptoms was dose-dependent (94). Moreover, adrenocortical degeneration seemed to be associated with a decrease in mitochondrial and lipid droplets number and an increase in lysosomes, autolysosomes, and lipid droplets dimension (94). Similar evidences were found in different animal models, including guinea pigs and cynomolgous monkeys (95, 96). Moreover, experimental studies on adrenocortical cell cultures derived from guinea pigs and clinical studies on guinea pigs showed that ATR-101 induced an impairment of mitochondrial respiration (95, 97). These findings were later confirmed in studies on ACC cell cultures, where mitochondrial membrane hyperpolarization, induction of apoptosis, increased reactive oxygen species, and reduced adenosine triphosphate (ATP) levels were observed after incubation with ATR-101 (91, 98, 99). Recently, a decrease in adrenal cholesterol efflux in ACC cell cultures in presence of ATR-101 was also reported, suggesting a potential new mechanism involving cholesterol transmembrane transport in the cytotoxic increase of intracellular free cholesterol (91).

Due to its adrenolytic effects, ATR-101 has been recently proposed as a new treatment for CS. In the last 5 years, two studies on animal models evaluated its effects on cortisol secretion (99, 100). In a study on three female dogs, ACTH-stimulated cortisol levels were reduced by 62 and 71% after a week of ATR-101 treatment at 3 mg/kg/day and after an additional week of ATR-101 treatment at 30 mg/kg/day, respectively. In addition, the ACTH-stimulated levels of the cortisol precursors 17OH-progesterone and 11-deoxycortisol were reduced after 2 weeks of ATR-101 treatment, and similar decrease occurred in ACTH-stimulated cortisol precursors corticosterone and 11-deoxycorticosterone, as well as the androgens androstenedione and dehydroepiandrostenedione sulfate (DHEAS) levels (99). In a proof-of-concept study on 10 dogs with endogenous CS, including seven dogs with CD, ATR-101 treatment at 3 mg/kg/day for 1 week, followed by 30 mg/kg/day for 1–3 weeks, showed to reduce ACTH-stimulated cortisol levels in nine (90%) of 10 cases. In particular, all seven (100%) dogs with CD reduced ACTH-stimulated cortisol levels, with a mean decrease of 49.8%. Considering the overall cohort, a significant decrease in ACTH-stimulated cortisol levels was identified after the first week of treatment and was maintained throughout the 4 weeks of study (100). Seven (70%) cases improved, two (20%) remained stable, and the remaining one (10%) worsened. A significant increase in ALT levels was observed together with a similar, albeit non-significant, increase in alkaline phosphatase (ALP) levels, and a mild decrease in hematocrit and serum albumin, which nevertheless remained within the normal range throughout the study; moreover, four episodes of vomiting and one episode of diarrhea were reported as AEs (100).

ATR-101 was investigated in a phase I, multicenter, open-label, ascending multiple-dose cohort study in adults with advanced ACC who had failed or declined previous therapy, but the whole cohort of patients discontinued treatment before study completion, mainly because of disease progression (51% of cases). Moreover, safety profile mainly showed gastrointestinal disturbances (101).

No evidences about ATR-101 treatment in human CD or CS patients are currently available. However, a phase II, randomized, double-blind, placebo-controlled, multicenter study (clinicaltrials.gov code: NCT03053271) to evaluate the efficacy and safety of ATR-101 treatment in CS patients was started on February 2017. In this study, four patients with CS and UC ranging from 1.3- to 10-fold the ULN entered an open-label, intra-subject, dose-escalation period of 8 weeks, followed by either a double-blind, randomized, withdrawal period of 4 weeks or an additional open-label dosing period of 4 weeks, according to randomization criteria. The primary endpoint of this study was the proportion of patients with either normalization or a ≥50% decrease in UC after 85 days of ATR-101 treatment. The study was closed on August 2019 due to slow enrollment. No preliminary, nor definitive results are currently available, therefore it is not possible to draw suggestions on its use in the clinical setting.

### Mitotane

Mitotane is an oral adrenolytic agent with adrenostatic properties represented by steroidogenesis inhibition; it is currently approved as a treatment for ACC, but also authorized as a treatment for severe CS in some European countries, including Italy since 2000.

Mitotane induces a cytotoxic effect on the mitochondrial system of adrenocortical cells, resulting in mitochondrial disruption, swelling, and lysis, followed by cell death (4). Moreover, mitotane inhibits several adrenal enzymes involved in adrenal steroidogenesis, including cholesterol side-chain cleavage complex, 11β-hydroxylase, 18-hydroxylase, and 3β-hydroxysteroid-dehydrogenase. Therefore, mitotane reduces cortisol production and secretion in two different combined mechanisms (4). Although having a long half-life, ranging from 18 days to about 5 months (4, 102), mitotane has a slow onset of action, potentially requiring up to 3 months to reach the therapeutic range (4, 102), therefore needing a daily administration on a twice-daily or thrice-daily schedule and a constant, monthly monitoring of its circulating levels to assess the therapeutic range. Mitotane is formulated as an immediate-release tablet, containing a 500 mg dose, and a maximum total daily dose of 6 g is usually recommended, although clinical monitoring of mitotane circulating levels is required to tailor the required dose for the single patient needs, in terms of efficacy and safety (4).

Being one of the first medical therapies to be evaluated in CS treatment during the 1960s, mitotane has shown high efficacy in treatment of CS, with a revised estimated average remission rate of 79.8% according to a recent meta-analysis (61). In particular, in studies including both exclusively CD and mixed CD and CS populations, mitotane showed an average remission rate of 86.9%, ranging from 72 to 100% of patients (4). Noteworthy, contrary to the classical exclusively adrenostatic agents, no treatment escape was reported during mitotane treatment in both CD and CS patients. In both CD and CS patients mitotane treatment is associated with a general improvement in clinical features, mainly overweight or obesity and impairment of glucose metabolism (4, 60). In CD patients, the effect of mitotane treatment on pituitary tumor was reported in a French study on 76 CD patients, where in 12 patients with no evidence of pituitary lesions at MRI, a pituitary tumor became apparent after a median time of 10.9 months after treatment start (4, 103). In studies including both exclusively CD and mixed CD and CS populations, the most frequently reported AEs were dyslipidemia (63.3%), gastrointestinal disturbances, including nausea, vomiting, and diarrhea (46.3%), and neurological disorders (29.9%) (4). In CD men, gynecomastia is also reported (17.6–50%), as mitotane also acts on gonadal steroidogenesis, impairing testicular androgen production (4, 60). Moreover, the adrenolytic action of mitotane usually leads to AI, requiring therefore glucocorticoid replacement, although the AI may be reversible after mitotane withdrawal (4, 60); particularly, a recent study on ACC patients exposed to mitotane in long-term treatments (at least 24 months) has shown that hypothalamus-pituitary-adrenal axis recovery may be present in around 78% of patients, with a mean time to recovery of 2.7 years (104). Despite the relevant number of CD patients treated with mitotane, all the available studies on mitotane as a treatment for CD are single-center, non-controlled studies, and two of the oldest studies included also patients undergone pituitary irradiation, potentially biasing the overall results (105, 106). Moreover, only one French study has been performed on the use of mitotane in CD treatment in the last 20 years (103), and therefore it cannot be excluded that the efficacy rates reported in the oldest studies may have been influenced by the reliability of the hormone assays available at that time, undoubtedly lower than that of the currently available hormone assays. Therefore, an international, multicenter, controlled study on mitotane as CD treatment is currently lacking.

Overall considered, mitotane seems to be an effective treatment in CD and CS, and its high potency may lead to consider it as a potential treatment in patients failing to respond to different treatment approaches. However, mitotane does not affect the pituitary tumor, which should be properly monitored in case of long-term treatment. Moreover, due to the poor safety profile, a careful monitoring of circulating levels of mitotane should be performed to avoid excessive levels and potential toxicity. In particular, due to the frequent occurrence of AI, clinical picture should be carefully monitored especially in the first months of treatment, and preventive glucocorticoid replacement should be performed during mitotane titration period. Furthermore, as monthly monitoring of mitotane circulating levels is required to assess its therapeutic efficacy, mitotane treatment should be preferred in patients with high adherence to routinely clinical evaluations, to avoid potential toxic effects related to mitotane overtreatment. Finally, because of long-lasting teratogenic effects, mitotane should be avoided in women seeking for pregnancy or pregnancy should be discouraged in women not only during treatment, but also in the period following mitotane treatment.

## Glucocorticoid Receptor-Directed Drugs

GR-directed drugs, or GR antagonists, target GR, impairing cortisol-GR binding and, therefore, reducing the peripheral effects of cortisol excess. Therefore, they aim not to normalize cortisol secretion and consequently control hypercortisolism-related signs, symptoms, and comorbidities, but to directly act on the clinical burden of chronic hypercortisolism (4). Considering currently available drugs, mifepristone was recently evaluated in clinical trials, whereas an experimental drug, relacorilant, is currently under investigation.

Table 4 shows the characteristics of current and recent clinical trials, registered in clinicaltrials.gov, on GR antagonists for the treatment of CD and CS in terms of official study name, identification code, study design, patient number, start, and completion date.

**Table 4:** Characteristics of current and recent clinical trials, registered in clinicaltrials.gov, on glucocorticoid-receptor antagonists for the treatment of Cushing's disease and Cushing's syndrome in terms of official study name, identification code, study design, patient number, start, and completion date.

**Investigation drug**	**Official study name**	**ClinicalTrials.gov code**	**Study type**	**Estimated patient number**	**Start date**	**Completion date**
Mifepristone	An Open-label Study of the efficacy and safety of CORLUX (mifepristone) in the treatment of the signs and symptoms of endogenous Cushing's syndrome	NCT00569582	OL; P; MC; Phase III	50*	December 2007	January 2011
	An Open Label Extension Study of the efficacy and safety of CORLUX (mifepristone) in the treatment of the signs and symptoms of endogenous Cushing's syndrome	NCT00936741	OL; P; MC; Phase III	30*	July 2009	September 2012
Relacorilant	Phase 2 Study of the safety and efficacy of CORT125134 in the treatment of endogenous Cushing's syndrome	NCT02804750	OL; P; MC; Phase II	35*	June 2016	September 2018
	Glucocorticoid receptor antagonism in the treatment of Cushing syndrome (GRACE): A Phase 3, Double-Blind, Placebo-Controlled, Randomized-Withdrawal Study of the efficacy and safety of relacorilant	NCT03697109	RW; DB; P; MC; Phase III	130	October 2018	November 2021

*OL, open-label; P, prospective; MC, multicenter; RW, randomized-withdrawal; DB, double-blinded*.

* *Definitive patient number due to study completion*.

### Mifepristone

Mifepristone is an oral non-selective GR antagonist, officially approved in the United States in February 2012 for the treatment of CS patients who have type 2 diabetes mellitus or glucose intolerance and have failed surgery or are not candidates for surgery. In Europe, no official approval has been granted to mifepristone as treatment for CS.

As a GR antagonist, mifepristone directly acts on cortisol-GR binding, thus not reducing cortisol secretion, but only its peripheral effects. Therefore, after mifepristone administration, a rise in circulating cortisol levels may be observed, as the GR blockade also has central effects, thus inducing a compensatory response by the hypothalamus-pituitary-adrenal axis and further driving corticotropin release hormone (CRH), ACTH, and cortisol secretion (4). However, the excessively high cortisol levels may lead to the activation of mineralocorticoid receptor due to a spillover effect, potentially causing hyperaldosteronism-like AEs, including hypertension, hypokalemia, and peripheral oedema (4). Likewise, an excessive GR blockade may also occur at high mifepristone doses, potentially leading to a clinical syndrome resembling AI, that is more appropriately identifiable as cortisol withdrawal syndrome (4). Moreover, being a non-selective steroid receptor antagonist, mifepristone also binds androgen and progestin receptors, inhibiting their peripheral effects; in particular, the antiprogestin effects may represent a safety concern, as endometrial thickening and abnormal vaginal bleeding may occur in women with CS treated with mifepristone (4). Finally, as the pharmacodynamic mechanism of GR antagonism does not allow to rely on biochemical markers of CS due to the compensatory rise in circulating cortisol levels, clinicians should evaluate treatment efficacy by monitoring hypercortisolism-related clinical syndrome and comorbidities, an approach uneasy to perform in clinical practice (4). Mifepristone has a long half-life (24–90 h), allowing therefore a once-daily administration (4). Mifepristone is formulated as an immediate-release capsule, containing a 300 mg dose, and a maximum total daily dose of 1,200 mg is currently suggested.

Mifepristone was initially conceived as a pro-abortive treatment, due to its antiprogestin effects, but it was later discovered to have an inhibitory effect on GR at higher doses (107). Therefore, since 1985, it has been used as an off-label treatment in selected cases, mainly represented by patients with severe ectopic CS, although only in the last 15 years, evidences from clinical studies and clinical trials have been published (4, 108, 109). In a European, multicenter, retrospective study on 20 CS patients, including four CD patients, mifepristone at doses of 600–1,200 mg/day induced improvement of clinical features in 15 (75%) patients, being particularly effective on the improvement of psychiatric symptoms and on the control of glucose metabolism. The most frequently reported AEs were hypokalemia (55%), hypertension (15%), and AI (15%) (108). Some years later, mifepristone was further investigated in a multicenter, open-label, prospective clinical trial, the SEISMIC study (clinicaltrials.gov code: NCT00569582) for a period of 24 weeks (109). Mifepristone was orally administered in a once-daily schedule. In this study, 50 CS, including 43 CD, patients with UC > ULN, entered a single 24-week, open-label period. According to investigators judgment, starting at the dose of 300 mg/day, a dose up-titration to 600, 900, and 1,200 mg/day, the maximum allowed dose, at week 2, 6, and 10, respectively, was performed in case of lack of clinical improvement. The primary endpoints of this study were the rate of responder patients, considered as patients with diabetes or impaired glucose tolerance (IGT) experiencing a ≥25% decrease in glucose area under the curve (AUC) at oral glucose tolerance test (OGTT) and as patients with hypertension experiencing a ≥5 mmHg decrease in diastolic blood pressure, after 24 weeks of treatment. At end of the study, mifepristone induced an improvement of glucose metabolism impairment in 15 (60%) of 25 patients with diabetes or IGT and an improvement of hypertension in eight (38.1%) of 21 hypertensive patients (109). Noteworthy, a clinical improvement was observed in 43 (87%) patients (109), with a decrease in body weight, waist circumference, and body fat, and an increase in insulin sensitivity (109–111). In a subsequent study, a follow-up analysis and long-term extension of SEISMIC study (clinicaltrials.gov code: NCT00936741), focused on the evaluation of change in body weight in 29 CS patients, including 26 with CD, a persistent and constant decrease in body weight was observed throughout the study, with 15 (83.3%) of 18 and eight (80%) of 10 patients with a weight loss of 5 and 10%, respectively, in the core study, maintaining or improving their weight during the extension study (112). Regarding the core study, considering the 16 CD patients with evidence of pituitary tumor at baseline pituitary MRI and with both baseline and 24-week pituitary MRI assessment, no significant change in tumor volume was observed. However, one additional patient with evidence of pituitary tumor at baseline pituitary MRI showed an increase in tumor volume at week 10, which led to mifepristone discontinuation (109). The most frequently reported AEs in the core study were nausea (48%), fatigue (48%), and headache (44%). AEs related to mineralocorticoid receptor activation included hypokalemia (44%), peripheral oedema (26%), and hypertension (24%). Moreover, 10 (28.6%) and five (14.5%) of the 35 women developed endometrial thickening and abnormal uterine bleeding, respectively, and in three (8.6%) patients, uterine dilatation and curettage was required to manage uterine disorders. Treatment discontinuation occurred in 16 (32%) patients, because of AEs in seven (14%). Noteworthy, no treatment discontinuation because of lack of efficacy occurred (109). Additional AEs were reported in the extension study: the more frequently reported were nausea (52%), hypokalemia (48%), and fatigue (45%). Moreover, endometrial thickening occurred in seven (35%) of the 20 women (112). Considering both the European study, the SEISMIC study, and its extension, AI was reported as AE in three (15%), two (4%), and five (17.2%) patients, respectively (108, 109, 112).

Overall considered, mifepristone represents an effective treatment for CD clinical syndrome and comorbidities, and its rapid action may lead to consider it as an interesting approach in patients requiring prompt relief from hypercortisolism-related symptoms and signs and/or comorbidities. In particular, mifepristone mainly improved glucose metabolism, making it an interesting choice for diabetic patients, as well as for patients with IGT or impaired fasting glucose. Moreover, the once-daily oral administration may be comfortable for patients, potentially improving patients' compliance, with a potential positive impact on success rate. However, mifepristone does not affect the pituitary tumor, which should be properly monitored in case of long-term treatment. As uterine disorders occurred in women, mifepristone should be preferred in men and in women with clinical history of hysterectomy, whereas uterine ultrasound monitoring should be performed in women without clinical history of hysterectomy. Likewise, due to the occurrence of hyperaldosteronism-like AEs, mifepristone should be preferred in patients not already experiencing hypokalemia or severe hypertension, and both potassium supplementation and antihypertensive treatment should be optimized before starting treatment. Finally, because of potential teratogenic effects, mifepristone should be avoided in women seeking for pregnancy.

### Relacorilant

Relacorilant is a novel oral GR antagonist, currently under clinical evaluation for CS treatment (5).

The research for new GR antagonists with a more tolerable safety profile was started after the evidences of the SEISMIC study on mifepristone, where the main AEs seemed to be related to the low selectivity of mifepristone in antagonizing steroid receptors (4, 109). Therefore, a new GR antagonist, relacorilant, was developed to overcome this limitation of mifepristone (113). Experimental studies showed that relacorilant was selective for GR without significant affinity for progestin receptor, therefore potentially able to reduce the AEs related to the antiprogestin effects typical of mifepristone (113). Moreover, relacorilant was tested in a rat model of exogenous CS, identifying a significant positive effect on glucocorticoid-induced impairment of glucose metabolism, which was similar to those achieved after mifepristone administration (113). In a phase I study on healthy subjects, relacorilant showed to be rapidly absorbed, with a half-life of about 11–19 h, allowing therefore a once-daily administration, although a delay in absorption was observed after food intake, and a good safety profile, with gastrointestinal disorders being the most frequently reported AEs after both single dose and multiple doses administration and musculoskeletal disorders essentially reported after multiple doses administration (114).

A phase II, multicenter, open-label clinical trial (clinicaltrials.gov code: NCT02804750), performed for a period of 20 weeks, was started in June 2016 to assess the efficacy and safety of relacorilant treatment in CS patients, and was completed by September 2018. Preliminary data have been recently presented in endocrinological meetings (115). Relacorilant was orally administered in a once-daily schedule. According to the starting dose, 35 CS patients, including 28 patients with ACTH-dependent CS, with UC > ULN were divided into two cohorts: in the first one (low dose, LD, group), starting dose was 100 mg/day, whereas in the second one (high dose, HD, group) starting dose was 250 mg/day. During up-titration period, a maximum dose of 200 and 400 mg/day in LD and HD patients, respectively, was reached. In this study, patients entered a 12 to 16-week, open-label, up-titration period, during which a dose up-titration was performed every 4 weeks, followed by a 4-week, open-label, stable-dose period. After the stable-dose period, relacorilant was discontinued and patients were followed-up after 4 weeks. The primary endpoints of this study were the response in glucose tolerance, defined as a decrease in HbA1c ≥0.5%, normalization or a ≥50 mg/dl decrease in 2-h glucose levels after OGTT, or a decrease in total daily insulin dose of ≥25% or in daily sulphonylureas dose of ≥50%, compared to baseline, and in blood pressure, defined as a decrease of ≥5 mmHg in either mean systolic or diastolic blood pressure levels compared to baseline, at study completion. After 4 weeks of treatment at stable doses, relacorilant induced an improvement in hypertension in five (41.7%) of 12 LD and seven (63.6%) of 11 HD patients, whereas an improvement in glucose metabolism impairment was observed in two (15.4%) of 13 LD and six (50%) of 12 HD patients regardless of dose increase (115). Moreover, a decrease in body weight was observed in six (35.3%) of LD and nine (60%) of HD patients, and an overall improvement in quality of life, depressive status, and neurocognitive function was reported (115). Regarding safety profile, the most frequently reported AEs were back pain (31.4%), headache (25.7%), and peripheral oedema (25.7%), with a higher prevalence in HD compared with LD patients. In HD patients, five serious AEs, including pilonidal cyst, myopathy, polyneuropathy, myocardial infarction, and hypertension, were reported. Noteworthy, no cases of hypokalemia or vaginal bleeding were observed, neither treatment discontinuation occurred (115). Definitive data are not yet available.

Currently, a phase III, multicenter, double-blind, placebo-controlled, randomized-withdrawal clinical trial, the GRACE study (clinicaltrials.gov code: NCT03697109), to assess the efficacy and safety of relacorilant treatment in CS patients for a period of 38 weeks is presently on-going. Relacorilant is orally administered in a once-daily schedule. This study expects to enroll 130 CS patients, which enter a first open-label, up-titration period for about 22 weeks, followed by a randomization period of 12 weeks, during which they are randomly and double-blindly assigned to relacorilant treatment or placebo. Patients start treatment at a 100 mg/day dose, that is up-titrated every 4 weeks until 400 mg/day or to maximum tolerated dose, depending on patients' response. At the end of the randomized period, both placebo and relacorilant treatments are withdrawn and patients enter a 4-week, follow-up period. The primary endpoints of this study are the glucose intolerance and hypertension, as compared between relacorilant and placebo treatments evaluated at the end of the randomization period in patients who met any response criteria in glucose intolerance and/or hypertension at the start of the randomization period, and the safety profile throughout the study. In detail, the endpoint in patients with diabetes mellitus/IGT with an initial response, including normalization or ≥50 mg/dL decrease of 2-h glucose levels after OGTT, decrease of ≥0.5% of HbA1c and/or decrease ≥25% of total daily insulin dose or ≥50% daily sulfonylurea dose, was the mean change in glucose AUC during OGTT, whereas the endpoint in patients with hypertension was the proportion of patients with loss of response with respect to hypertension, defined as an increase in systolic and/or diastolic blood pressure of ≥5 mmHg at ambulatory blood pressure monitoring or any increase or modification in antihypertensive medication. Expected study completion date is November 2021, but no preliminary data are available.

Overall considered, relacorilant seems to be a promising effective treatment for the control of CD, and CS, clinical syndrome and comorbidities, in particular for patients with hypertension or glucose metabolism impairment, with a good safety profile and a comfortable once-daily oral administration; however, definitive data are required to draw definite conclusions on its potential use in the clinical setting.

## Combination Therapy

Combination therapy represents a valuable, although not extensively studied, option for the treatment of CD and CS, as no actually available therapy is able to reach a 100% efficacy in normalizing cortisol levels, as well as in reverting hypercortisolism-related clinical features and comorbidities. Indeed, combination of two or more drugs may theoretically improve the antisecretory efficacy of single treatments, allowing therefore a potential use of lower doses for each drug and, therefore, hypothetically reducing the rate of treatment-related AEs without losing therapeutic efficacy. Up to date, four studies have been published on combination therapy in CD and/or CS patients, both using drugs acting at the same level (116) and at different levels (55, 56, 117). In a study on adrenal-directed agents on 11 severe CS patients, including four CD, combination of ketoconazole (400–1,200 mg/day), metyrapone (3–4.5 g/day), and mitotane (3–5 g/day) induced UC normalization (UC ≤ ULN) in seven (63.6%) patients after 24–48 h, with UC remaining low to normal throughout the period of combination therapy, with improvement of clinical status. The most frequently reported AEs were hypokalemia (100%), mainly initially experienced as episodes, increase in GGT (81.8%), and nausea and vomiting (63.6%) (116). In a study combining pasireotide (300–750 μg/day), cabergoline (1.5–4.5 mg/week), and ketoconazole (600 mg/day) treatments on 17 CD patients, the combination therapy induced UC normalization (UC ≤ ULN) in 15 (88.2%) patients, using a stepwise approach with pasireotide as the starting treatment and cabergoline and ketoconazole as first and second additional treatment, respectively, in case the previous first-line or second-line treatment failed to normalize UC at maximum doses (117). An improvement of clinical picture was observed, with a decrease in body weight, waist circumference, and blood pressure. The most frequently reported AEs were disturbance of glucose homeostasis and serum insulin-like growth factor 1 (IGF1) levels decrease below the normal range (52.9%) (117). Combined treatment with cabergoline and ketoconazole was investigated in a Brazilian study on nine patients with persistent CD after surgery (55) and in an Italian study on 14 patients with persistent or recurrent CD after surgery or without prior surgery (56). In the Brazilian study, the addition of ketoconazole (200–400 mg/day) to cabergoline (3 mg/week) in nine patients unable to normalize UC after 6 months of cabergoline monotherapy induced UC normalization (UC ≤ ULN) in six (66.7%) patients after 6 months, with a mild increase in liver enzymes in one (11.1%) patient after ketoconazole addition as the only reported AE (55). In the Italian study, the addition of ketoconazole (200–600 mg/day) in six patients treated with cabergoline (0.5–3 mg/week), which was unable to normalize mUC and LNSC levels after 6 months of cabergoline monotherapy, induced mUC normalization in five (83.3%) patients after 6 months, whereas the addition of cabergoline (0.5–3 mg/week) in eight patients treated with ketoconazole (200–600 mg/day), which was unable to normalize mUC and LNSC levels after 6 months of ketoconazole monotherapy, induced mUC normalization in five (62.5%) patients after 6 months of combined therapy. An improvement of the clinical picture was observed, with a decrease in body weight, waist circumference, and HbA1c levels. Moreover, a drop in the number and/or dose of antihypertensive drugs, up to withdrawal, was also reported. Noteworthy, no additional AEs were observed during combined therapy compared with cabergoline or ketoconazole monotherapies (56). Although not specifically focused on combined therapy, a French retrospective, multicenter study on cabergoline treatment in CD also reported evidences of combined treatment with cabergoline and adrenal steroidogenesis inhibitors in nine patients in preparation for pituitary surgery (one patient) or after unsuccessful surgery (eight patients) (58). In this study, the addition of cabergoline (0.5–3.5 mg/week) to ketoconazole (600–1,200 mg/day) in seven patients and to metyrapone (3.75–6 g/day) in two patients was able to normalize UC in five (55.5%) patients during the first 12 months of treatment, with UC normalization within 6 months in two (22.2%) patients and within 8 months in the remaining three (33.3%) patients. An improvement in the clinical picture was observed, with a decrease in body weight, glucose metabolism, and blood pressure (58). AEs occurring during combined therapy were not specifically reported in this study.

A phase II, open-label, multicenter, worldwide clinical trial on the combination of two pituitary-directed drugs, pasireotide, and cabergoline, the CAPACITY study (clinicaltrials.gov code: NCT01915303), was started to assess the efficacy and safety in CD patients. Started in March 2014 and completed on September 2019, preliminary data have been presented in endocrinological meetings (118) and more recent data have been provided on clinicaltrials.gov. Pasireotide was administered in its classical subcutaneous formulation (1,200–1,800 μg/day), in a twice-daily schedule, whereas cabergoline was administered in an oral, once-daily schedule (0.5–1 mg/day). Overall, 68 CD patients with mUC > ULN, with or without prior surgery, pasireotide-naïve and who discontinued pasireotide after a previous treatment for reasons other than AEs, entered a 35-week, open-label study, starting with an 8-week, stable-dose period of pasireotide at a total dose of 1,200 μg/day. At week 9, 18, and 27, patients with mUC > ULN underwent pasireotide up-titration to a total dose of 1,800 μg/day, addition of cabergoline at 0.5 mg/day, and cabergoline up-titration to 1 mg/day, respectively, whereas patients with mUC ≤ ULN continued the previously assigned treatment throughout the study. The primary endpoint of this study was the proportion of patients achieving mUC normalization (mUC ≤ ULN) with pasireotide monotherapy or in combination with cabergoline at week 35. Considering the overall population, 34 (50%) patients achieved mUC normalization after 35 weeks of treatment; in detail, 17 (25%) patients achieved mUC normalization with pasireotide monotherapy, whereas the remaining 17 (25%) required combination therapy. A decrease in body weight, waist circumference, blood pressure, and total cholesterol levels was observed, altogether with an overall improvement in clinical signs and quality of life. The most frequently reported AEs were hyperglycemia (51.5%), nausea (51.5%), and diarrhea (44.1%). Treatment discontinuation occurred in 16 (23.5%) patients, because of AEs in eight (11.8%) and lack of efficacy in three (4.4%) patients. Definitive data are not yet available.

Overall considered, combination therapy may represent an interesting approach in case of partial response or intolerance to monotherapy in patients with CD. Keeping in mind that normalization of cortisol levels is only one of the goals in the treatment of CD, as it should rely both on hormonal and tumor growth control, the combined use of adrenal-directed agents in CD, although highly effective in hypercortisolism control, is not useful for pituitary tumor growth control, and therefore may be an option especially in case of very severe CD requiring immediate relief from hypercortisolism-related comorbidities. Moreover, the association of two pituitary-directed drugs may improve the effects on tumor growth, although normalization of cortisol secretion may require time to be reached or may not be achieved, even with a combined approach. Conversely, the contemporary use of an adrenal-directed and a pituitary-directed agent, one of the most investigated in the limited available experiences, may combine the fast resolution of hypercortisolism granted by the adrenal-directed agents to the tumor growth control granted by the pituitary-directed agents. Similarly, an unexplored, although potentially feasible, approach may be represented by GR antagonists and pituitary-directed agents association, as the former may help in a more effective control of hypercortisolism-related comorbidities compared with monotherapy, whereas the latter may reduce the excessive ACTH secretion by the pituitary tumor and prevent tumor growth, or even favoring tumor shrinkage. In conclusion, although the combination of adrenal-directed and pituitary-directed agents seems to theoretically be the best approach, every combination therapy schedule should be considered and/or tested, ideally tailoring it to patient clinical features and disease characteristics, as well as to the pharmacological profile of the single agents, and to the effect of the potential combination in terms of efficacy and tolerability.

## Conclusions

Nowadays, medical therapy represents a relevant option in the treatment of patients with CD, particularly in those with persistent or recurrent CD, and especially when the alternative second-line treatments, including repeat pituitary surgery, pituitary radiotherapy, and bilateral adrenalectomy, are not feasible, not indicated or not preferred by patients. However, currently available and routinely used drugs have their pitfalls, leading therefore to the research of new compounds or new formulations of already known compounds. In the recent years, many clinical trials on the medical treatment of CD started, showing promising results in terms of disease control and safety profile. Among pituitary-directed agents, the introduction of pasireotide LAR, with its intramuscular once-monthly administration, may reduce the discomfort associated with the subcutaneous formulation and improve medical adherence, although it has shown similar efficacy and safety. Indeed, hyperglycemia-related AEs still represent a major issue in pasireotide treatment, independently of the pharmacological formulation. Two new steroidogenesis inhibitors, osilodrostat, and levoketoconazole, have shown promising results in recently completed clinical trials. In particular, osilodrostat seems to offer a fast, sustained, and effective UC normalization in a large number of patients, although cortisol syndrome or AI, and adrenal hormone precursors-related AEs, mainly including peripheral oedema and hypokalemia, may occur. Likewise, levoketoconazole may offer an alternative to classical ketoconazole treatment due to its hypothetical higher potency and potential lower hepatotoxicity, but its efficacy and safety profile is yet to be definitively assessed. Moreover, the efficacy in CS treatment of metyrapone, a classical adrenal steroidogenesis inhibitor, will hopefully be clarified by the on-going trial, offering a prospective, multicenter, international experience. Considering GR antagonists, relacorilant has shown interesting results in improving hypertension and impairment of glucose metabolism, with a safety profile apparently better than mifepristone, the only actually available GR antagonist, although further evidences from wider phase III studies are required. Among the most recently evaluated drugs, roscovitine and ATR-101 may offer new therapeutic options in the next future, according to the available preclinical evidences, but data from human patients are mandatory to definitively evaluate their potential impact on CD treatment. Finally, combination therapy could be considered a valuable alternative in patients unresponsive to a single drug treatment, even if further larger studies are needed to assess the efficacy and safety of a polypharmacotherapy for CD treatment, especially for unexplored combinations, such as GR antagonists and pituitary-directed agents. In conclusion, the landscape of medical treatment for CD will be hopefully enriched in the next years by new, effective drugs with different therapeutic targets, therefore further helping clinicians in addressing the needs of each patient in a more tailored approach, in order to improve the therapeutic outcome and to reduce the burden of illness.

## Author Contributions

RP and RF structured the review. RF, MCDM, CS, ND, and LB performed the literature review. MN and CD provided update and poster abstracts from endocrinological meetings. MCDM, CS, and CP performed the interim review. RP and AC performed the final review and overall surveillance on the writing process. All authors contributed to the article and approved the submitted version.

## Conflict of Interest

RP has been Principal Investigator of Research Studies for Novartis, Recordati, HRA Pharma, Ipsen, Shire, Corcept Therapeutics, Cortendo AB; Co-investigator of Research Studies for Pfizer; received research grants from Novartis, Pfizer, Ipsen, HRA Pharma, Shire, IBSA; has been occasional consultant for Novartis, Recordati, Ipsen, Pfizer, Shire, HRA Pharma, Corcept Therapeutics, Cortendo AB, Bresmed, Ferring and Italfarmaco; and has received fees and honoraria for presentations from Novartis, Recordati, Shire. AC has been Principal Investigator of Research Studies for Novartis, Ipsen, Pfizer, and Lilly; Co-Investigator of Research Studies for Merck and Novo Nordisk; has been occasional consultant for Novartis, Ipsen, Pfizer, and Italfarmaco; and has received fees and honoraria for presentations from Novartis, Ipsen, and Pfizer. The remaining authors declare that the research was conducted in the absence of any commercial or financial relationships that could be construed as a potential conflict of interest.
